# Clathrochelate Complexes Containing Axial Cymantrene and Tromancenium Moieties

**DOI:** 10.1002/ejic.202300368

**Published:** 2023-07-17

**Authors:** Reinhard Thaler, Holger Kopacka, Klaus Wurst, Thomas Müller, Florian R. Neururer, Stephan Hohloch, Petra Lippmann, Ingo Ott, Benno Bildstein

**Affiliations:** ^1^ Institut für Allgemeine, Anorganische und Theoretische Chemie Universität Innsbruck Innrain 80–82 6020 Innsbruck Austria; ^2^ Institut für Organische Chemie Universität Innsbruck Innrain 80–82 6020 Innsbruck Austria; ^3^ Institute of Medicinal and Pharmaceutical Chemistry Technische Universität Braunschweig Beethovenstr. 55 38106 Braunschweig Germany

**Keywords:** clathrochelates, cobalt, cytotoxicity, iron, manganese

## Abstract

New clathrochelate complexes of manganese, iron and cobalt containing peripheral organometallic manganese moieties cymantrene or tromancenium were synthesized via self‐assembly from di/tri‐topic dioximes, metal templates and cymantrene/tromancenium boronic acid pinacol esters. These air‐stable, highly colored, oligometallic complexes are composed of various combinations of Mn^I^Fe^II^Mn^I^, Mn^I^Co^II^Mn^I^, Mn^I^Mn^II^Mn^II^Mn^I^ and Mn^I^Co^II^Co^II^Mn^I^ metal assemblies with corresponding complicated magnetic and electrochemical properties. Full spectroscopic and structural characterization by ^1^H/^11^B/^13^C NMR, HRMS, IR, UV‐vis, single crystal XRD and CV (cyclic voltammetry) is provided. Tetrametallic complexes containing tromanceniumyl substituents with two Co^II^ or Mn^II^ central metals exhibit promising anticancer properties against different tumor cell lines.

## Graphical abstract

Clathrochelate complexes of Fe^II^, Co^II^ and Mn^II^ with endcapping organometallic cymantrene or tromancenium groups were synthesized, spectroscopically and structurally characterized as well as electrochemically analyzed. Antitumor properties were investigated, revealing promising properties in the case of tromancenium‐substituted species.

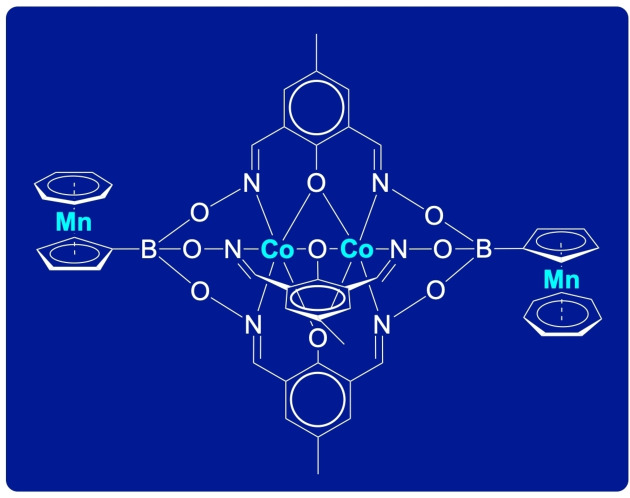

## Introduction

Clathrochelate complexes are cage coordination compounds formed by template condensation of suitable chelating ligands with metal ions.^[1]^ Well‐known and important members in this area of supramolecular chemistry are macropolycyclic tris‐dioximato complexes made from 1,2‐dioximes, boronic acids, and mostly Fe^II^ or Co^II^ metal centers.^[1]^ Structurally, these metal complexes contain boron‐capped octahedral to trigonal‐prismatic N_6_‐coordinated metal centers, depending on the size of the encapsulated metal center. Electronically, clathrochelate metal complexes have useful reversible redox properties with applications as electro/photo‐catalysts in the hydrogen evolution reaction from water^[2]^ or as abiotic biochemical and medicinal agents in diagnostics and therapy.^[3]^ Although the chemical space of clathrochelate complexes is quite well developed,^[1]^ organometallic examples are rare and currently limited to various ferrocene derivatives that are mainly of interest as redox‐responsive oligometallic systems.[^1a^, ^1b^]

Here we report on new tri or tetrametallic cage complexes of Mn^II^, Fe^II^ or Co^II^ ions containing the organometallic Mn^I^ boron‐capping moieties cymantrene or tromancenium, (*η* ^7^‐cycloheptatrienyl)(*η* ^5^‐cyclopentadienyl)manganese, based on our recent exploration of tromancenium chemistry.^[4]^ Synthetic aspects, structural and spectroscopic characterization, electrochemical as well as anticancer properties are presented.

## Results and Discussion

### Synthesis

**Scheme 1:**
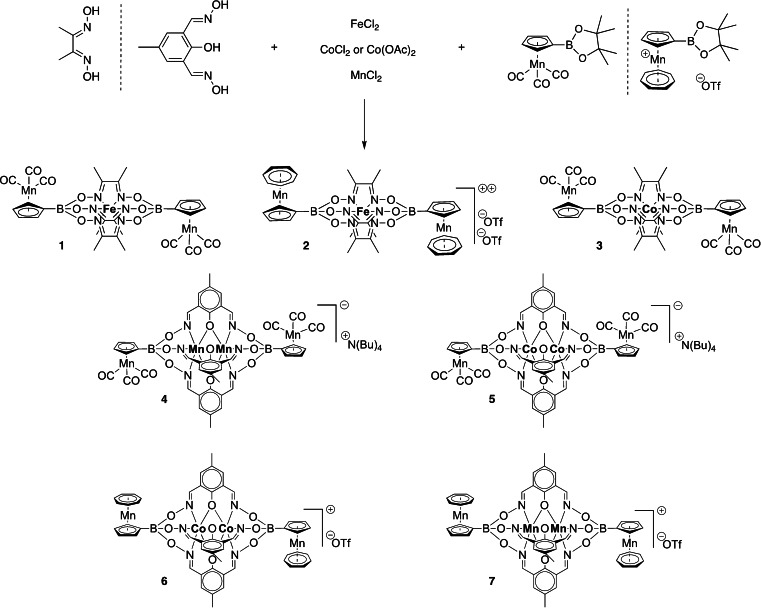
Synthesis of complexes **1**–**7**.

In general, clathrochelate complexes are easily synthesized in a one‐pot reaction without the exclusion of air at ambient conditions from simple precursors by metal‐templated condensation between di or tritopic dioximes and boronic acids (Scheme 1). Historically, most common is the use of simple 1,2‐dioxime ligands like dimethylglyoxime resulting in N_6_‐caged monometallic Fe^II^ or Co^II^,^[1]^ whereas with the much less popular starting material 2‐hydroxy‐5‐methylisophthalaldehyde dioxime further interesting N_3_O_3_N_3_‐caged bimetallic Mn^II^/Mn^II^ and Co^II^/Co^II^ complexes are accessible.[^1c^, ^5^] In this work, we employ the new organometallic Mn^I^ boronic acids cymantrenylboronic acid^[4b]^ or tromanceniumylboronic acid^[4b]^ as axially capping moieties that introduce two additional Mn^I^ metal centers, thereby giving access to trimetallic (**1**, **2**, **3**) or tetrametallic (**4**, **5**, **6**, **7**) complexes, respectively (Scheme 1). For simplicity and synthetic reasons^[4b]^ we used the easily hydrolyzed pinacolato esters^[4b]^ of these boronic acids instead of the free boronic acids (see Experimental Section). The isolated chemical yield of these metal‐templated condensation reactions is in the range of 15–85 %, depending on combination of constituents and charge and polarity of the complexes. Note that only cymantrenyl‐substituted complexes **1** and **3** are uncharged, whereas all other complexes are either mono/di‐cationic (**2**, **6**, **7**) or mono‐anionic (**4**, **5**). Also note that a likely bis‐tromanceniumyl‐tris‐glyoximato−Co^II^ complex is missing in this series because this material proved unstable towards air and chemical yields were quite low, a general feature in clathrochelate chemistry where condensation reactions work much better with Fe^II^ than with Co^II^ templates.^[1]^ In principle, other metal centers might form analogous cage complexes, but a few attempts with Ni^II^ or Zn^II^ templates gave in our hands no positive results.

### Structural, Spectroscopic, Electrochemical and Biological Properties

Clathrochelate complexes **1**–**7** are air‐stable compounds with melting or decomposition points ranging from 208–300 °C, in line with their high molar mass and mostly ionic nature. High‐resolution mass spectrometry gave proof of their identity in very good agreement of experimental and calculated values of their most abundant monoisotopic peaks. Elemental analyses of **1**–**5** are in acceptable congruence of theoretical and experimental data, except for tromaceniumyl‐substituted tetrametallic complexes **6** and **7** that afforded only poor data despite repeated combustion analyses.

Characterization by NMR spectroscopy is only possible for diamagnetic complexes **1** and **2** that contain low‐spin d^6^ metal centers Mn^I^ and Fe^II^, whereas **3**–**7** are paramagnetic d^7^ (Co^II^) or d^5^ (Mn^II^) species. The two iron complexes **1** and **2** show ^1^H/^13^C/^11^B NMR signal patterns in line with their structural subunits and‐regarding the axial cymantrene and tromancenium moieties‐similar to other such manganese (half)sandwich complexes.^[4]^ An unusual feature of simple sufficiently symmetric cymantrene and tromancenium compounds is the observation of resolved ^55^Mn NMR signals despite the high quadrupolar moment of ^55^Mn.^[4]^ However, no ^55^Mn NMR spectra for **1** and **2** could be obtained, due to the high asymmetry induced by the bulky clathrochelate iron moiety.

IR spectroscopy of cymantrenyl‐substituted complexes **1**, **3**, **4** and **5** show the expected diagnostic strong CO absorptions at approximately 1900 cm^−1^, whereas weaker C=N bands at approximately 1600 cm^−1^ (partially overlapped with C=C stretching vibrations) are observable for all complexes **1**–**7** (compare Supporting Information).

Clathrochelate complexes **1**–**6** are highly colored yellow‐red materials that give rise to corresponding UV‐vis spectra with strong absorptions and molar absorption coefficients *ϵ* in the range of 2643–12993 L ⋅ mol^−1^ ⋅ cm^−1^, whereas mono‐cationic, acceptor‐substituted Mn_4_ complex **7** is a less colored, beige material (see Supporting Information).

The magnetic moment of paramagnetic complexes **3**–**7** was measured at room temperature by the Evans NMR method,^[6]^ indicating varied spin states (Table 1), ranging from low‐spin to high‐spin configurations with or without antiferromagnetic coupling. Further detailed magnetic susceptibility studies in comparison to other clathrochelate complexes[^5^, ^7^] are beyond the scope of this work.

**Table 1:** Magnetic properties of **3**–**7** at room temperature.

compound	*μ*_B_ (Bohr magneton)	unpaired electrons	proposed spin state
**3**	3.92 (exp) 3.87 (calcd)	3	high‐spin Co^II^
**4**	11.25 (exp) 11.17 (calcd)	10	high‐spin Mn^II^Mn^II^
**5**	3.20 (exp) 2.83 (calcd)	2	low‐spin Co^II^Co^II^
**6**	6.80 (exp) 6.99 (calcd)	6	high‐spin Co^II^Co^II^
**7**	5.84 (exp) 5.92 (calcd)	5	high‐spin Mn^II^Mn^II^ with weak antiferromagnetic coupling

**Figure 1:**
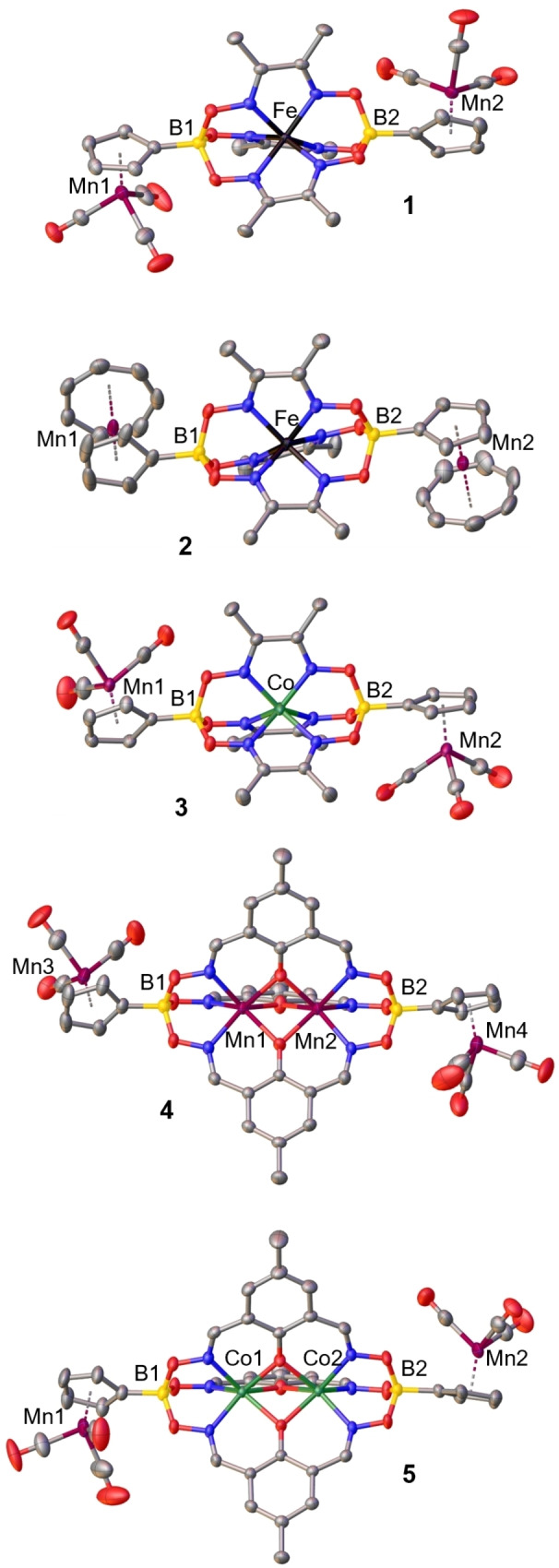
Molecular structures of complexes **1**–**5**. Hydrogen atoms and counterions are omitted for clarity. Thermal ellipsoids are shown with 50 % probability.

Single crystal X‐ray structure analyses are available for **1**–**5** (Figure 1) but not for tromanceniumyl‐substituted **6** and **7**, which provided only unsuitable crystals despite many efforts. Overall, the molecular structures prove the identity of these clathrochelates with structural metrics in line with expectations. Table 2 summarizes pertinent bond distances and angles. The coordination sphere at the sixfold coordinated central metals is more or less trigonal prismatic with twist angles *φ* ranging from 1.17° to 8.80 (trigonal prismatic coordination: *φ*=0°; octahedral coordination: *φ*=60°), a common situation in clathrochelate complexes due to the geometric restraints of the chelating oximato ligands.[^1^, ^8^] Metal−metal distances Mn−Mn and Co−Co of bimetallic complexes **4** and **5** are >2.9 Å, ruling out direct metal−metal bonding. The axial tromanceniumyl substituents of **2** have averaged cyclopentadienyl and cycloheptatrienyl Mn−carbon distances of similar value (Mn−C_av_≈2.12 Å), as has been also found in other tromancenium salts.^[4]^

**Table 2:** Pertinent distances [Å] and angles [°] of **1**–**5**.

compound	M−N_av_	M−O_av_	*φ*	M•••••M
**1**	1.91		8.22	
**2**	1.91		8.80	
**3**	1.97		1.17	
**4**	2.17	2.13	2.25	2.9041(6)
**5**	2.10	2.10	3.02	2.9646(6)

Oligometallic clathrochelate complexes **1**–**7** contain metal assemblies Mn^I^Fe^II^Mn^I^, Mn^I^Co^II^Mn^I^, Mn^I^Mn^II^Mn^II^Mn^I^ or Mn^I^Co^II^Co^II^Mn^I^. The expected rich redox chemistry (Table 3) is clearly evident from their cyclic voltammograms (see Supporting Information) with oxidation events based on Mn^I^/Mn^II^, Fe^II^/Fe^III^, Co^II^/Co^III^ and reduction events based on Mn^I^/Mn^0^ and Mn^II^/Mn^I^ couples, respectively. We observe various (quasi)reversible or irreversible electron transfers that are more detailed in Table 3. The first oxidation wave is due to the Mn^I^/Mn^II^ couple at more or less similar potentials as in other tromancenium salts,^[4]^ whereas the second oxidation refers to the oxidation of the central metal(s). Similarly, the first reduction event is due to the Mn^I^/Mn^0^ couple at comparable potentials as in other tromancenium salts,^[4]^ whereas the second reduction is most likely central metal based. Please note that generally in electrochemical measurements of tromancenium compounds^[4]^ significant electrode passivation occurs that sometimes hamper detection of cyclic voltammograms‐in this work no reliable data for complex **7** could be obtained. In comparison to other clathrochelate complexes reported in the literature,[^1^, ^8^] cymantrenyl and tromanceniumyl‐substituted clathrochelate complexes are electrochemically more interesting and complicated, due to their additional axial organometallic (half)sandwich Mn^I^ moieties.

**Table 3:** Redox potentials of clathrochelate complexes **1**–**6**.^[a]^

compound	E_ox,2_	E_ox,1_	E_red,1_	E_red,2_	further redox processes
**1**	n.o.	+0.75^[b]^	−1.74^[c]^	−2.24^[b]^	associated oxidized form gives rise to a follow−up chemical product which is reduced at −0.23 V
**2**	n.o.	+0.84^[b]^	−1.54^[b]^	−1.92^[b]^	associated oxidized form gives rise to follow‐up chemical products which are reduced at +0.68 V and −0.68 V, the reduced form gives rise to a species that is oxidized at −0.73 V and +0.10 V
**3**	+1.01^[b]^	−0.06^[c]^	−1.21^[c]^	−2.21^[b]^	associated oxidized form gives rise to a follow‐up chemical product which is reduced at −0.40 V
**4**	n.o.	+0.52/+0.40^[b]^	−2.22^[b]^	−2.60^[b]^	
**5**	+0.81^[b]^	+0.27^[b]^	−2.26^[b]^	n.o.	
**6**	+0.93^[b]^	+0.41^[b]^	−1.63^[b]^	−1.92^[b]^	additional wave at +0.71 V

[a] Cyclic voltammograms were recorded in CH_3_CN solution at a scan rate of 100 mV s^−1^. Potentials are given in volts and are calibrated against the FcH/FcH^+^ redox couple. [b] Peak potential of an irreversible redox event. [c] Half‐wave potential of an electrochemically quasi‐reversible or reversible process.

Redox‐active metal complexes are currently of topical interest as potential anticancer metallodrugs.^[9]^ Therefore we were curious how clathrochelate complexes containing organometallic (half)sandwich manganese(I) complexes **1**–**7** perform in this area. Table 4 shows the results of cytotoxicity studies against various standard cancer cell lines, namely A549 human lung carcinoma, HT‐29 human colon adenocarcinoma, human breast adenocarcinoma. VeroE6 monkey kidney epithelial cells were used to check for a possible tissue selectivity. Whereas unsubstituted tromancenium hexafluoridophosphate **A** ^[4a]^ and cymantrenyl‐substituted clathrochelate complexes **1**, **3**, **4**, **5** are inactive, tromancenium‐containing complexes **2**, **6** and **7** show quite promising antiproliferative effects. The effects obtained with VeroE7 cells were comparable to those with the cancer cells, indicating that the complexes do not show selectivity for cancer cells over non cancer cells. Notably, tetrametallic complexes **6** and **7** with more redox‐active metal centers than trimetallic complex **2** are the best candidates, emphasizing the importance of multiple redox processes in anticancer metallodrugs.^[9c]^ Further, it is interesting to note that the biological activity of the tromancenium‐containing complexes **2**, **6** and **7** depended on their combination with the clathrochelate unit, whereas such combination was not efficient in case of the cymantrene complexes **1** and **3**–**5**. The cytotoxicity data suggest further future studies, in particular on **6** and **7** and related complexes, in the emerging field of bioinorganic supramolecular coordination chemistry, which has considerable potential regarding molecular recognition, drug discovery or drug delivery amongst other areas.^[10]^

**Table 4:** Antiproliferative effects (IC_50_ values in μM) of **1**–**7**.

compound	A 549	HT‐29	MCF‐7	VeroE6
**A** ^[a]^	>100	>100	>100	>100
**1**	>80	>80	>80	>80
**2**	>12	9.82±0.81	10.86±0.54	>12
**3**	>50	>50	>50	>50
**4**	>100	>100	>100	>100
**5**	>50	>50	>50	>50
**6**	0.12±0.08	0.13±0.09	0.12±0.04	0.29±0.0
**7**	0.13±0.09	0.13±0.09	0.12±0.08	0.17±0.12

[a] **A**=parent unsubstituted tromancenium salt (*η* ^7^‐cycloheptatrienyl)(*η* ^5^‐cyclopentadienyl)manganese(I) hexafluoridophosphate^4a^

## Summary and Conclusions

Metal‐template assisted condensation of dioximato ligands with cymantrenyl or tromenceniumyl boronic acid pinacolato esters afforded clathrochelate complexes of Mn^II^, Fe^II^ and Co^II^ with axial Mn^I^ organometallic moieties. Depending on di‐ or tritopic dioxime building blocks, these new organometallic cage coordination compounds are either trimetallic/tetrametallic or diamagnetic/paramagnetic complexes with corresponding interesting electrochemical and magnetic properties. Structural, spectroscopic and cytotoxicity/anticancer studies are reported.

## Experimental Section

**General procedures**: Standard methods and procedures of organic/organometallic synthesis were performed. ^1^H, ^11^B, ^13^C and ^55^Mn NMR spectra were recorded at ambient temperature on a Bruker Avance DPX300 NMR spectrometer. Signals were referenced internally against ^1^H/^13^C residual solvent peaks or externally: ^11^B, B(OCH_3_)_3_ neat (for comparison of data to the IUPAC standard BF_3_ ⋅ Et_2_O+19.1 ppm has to be added). Mass spectrometric data were measured on a Thermo Finnigan Q Exactive Orbitrap spectrometer, IR spectra were recorded on a Bruker ALPHA IR spectrometer, UV‐vis spectra were measured on a PerkinElmer Lambda XLS+ spectrometer, single‐crystal X‐ray diffraction data were collected on a Bruker D8 Quest diffractometer with graphite‐monochromated Mo Kα radiation (λ=0.71073 Å) and structures were solved by direct methods. Cyclic voltammograms were recorded in an argon filled glovebox, using a BioLogic SP‐150 potentiostat with a three‐electrode setup (glassy carbon working electrode, platinum wire counter electrode, silver wire pseudo reference) and NBu_4_^+^PF_6_^−^ as supporting electrolyte (0.15 M). All potentials were calibrated internally to the ferrocene/ferrocenium redox couple. Due to significant electrode passivation, repeated electrode polishing was necessary in order to obtain reproducible results. Elemental analyses were obtained on an Elementar Analysensysteme GmbH Vario Micro Cube instrument.

**Starting materials**: Standard chemicals were obtained commercially and used as received. Cymantrenylboronic acid pinacolato ester,^[4b]^ tromanceniumylboronic acid pinacolato ester triflate^[4b]^ and 2‐hydroxy‐5‐methylisophthalaldehyde dioxime^[5]^ were prepared according to literature.

**Synthesis of 1**: A round bottom flask was charged with 11.5 mg of dry FeCl_2_ (1 equiv, 0.091 mmol) and dissolved in 10 mL of methanol. A color change from colorless to orange was observed when 31.6 mg (3 equiv, 0.272 mmol) of N,N′‐dihydroxy‐2,3‐butanediimine, also known as dimethylglyoxime, was added. A second color change from orange to red was observed when 60.0 mg of cymantrenylboronic acid pinacol ester (2 equiv, 0.181 mmol) was added. The mixture was allowed to stir over night while a red solid partially precipitated. The solvent was removed on a rotary evaporator. The product was dissolved in dichloromethane and filtered through a folded paper filter. Dichloromethane was removed in vacuo and the crude product was purified by short column chromatography on silica with diethyl ether as eluent affording **1** as diamagnetic red crystals in 64 % yield (48.1 mg, 0.058 mmol). Mp: 232 °C dec. ^1^H NMR (300 MHz, CD_2_Cl_2_, ppm) δ=2.40 (s, 18H, CH_3_), 4.74 (pseudo‐t, 4H, J=3.0 Hz), 5.06 (pseudo‐t, 4H, J=3.0 Hz). ^13^C NMR (75 MHz, CD_2_Cl_2_, ppm) δ=13.5 (CH_3_), 82.9 (C2/C5 of Cp), 90.3 (C3/C4 of Cp), 129.8 (ipso‐carbon of Cp), 152.8 (imine‐carbon), 226.8 (CO). ^11^B NMR (96 MHz, CD_2_Cl_2_, ppm) δ=‐12.95. IR (ATR, cm^−1^): 2957, 2924, 2855 (ν_C−H_), 2005, 1924, 1905 (ν_C=O_), 1581 (ν_C=N_), 1488, 1460, 1376 (ν_C=C_), 1268 (ν_B−O_), 1237, 1203 (ν_C−B_), 900 (ν_N−O_), 632 (ν_Fe_). HRMS (ESI pos, m/z) 825.9878, calc. for C_28_H_26_B_2_Mn_2_N_6_O_12_Fe: 825.9900. UV‐vis (CH_2_Cl_2_, [nm]): λ_max1_=280 (ϵ_1_=2912 L ⋅ mol^−1^ ⋅ cm^−1^), λ_max2_=442 (ϵ_2_=3581 L ⋅ mol^−1^ ⋅ cm^−1^). Anal. calculated for C_28_H_26_B_2_Mn_2_N_6_O_12_Fe: C 40.72, H 3.17, N 10.18; found: C 41.08, H 3.52, N 10.61. Single crystals were obtained by diffusion crystallization from acetone/pentane at 4° C. **1** is soluble in diethyl ether, dichloromethane, acetonitrile, acetone, dimethyl sulfoxide and insoluble in hydrocarbons like heptane.

**Synthesis of 2**: A round bottom flask was charged with 10.22 mg of FeCl_2_ ⋅ 4H_2_O (1 equiv, 0.051 mmol) and dissolved in 20 mL of methanol and 17.9 mg (3 equiv, 0.154 mmol) of dimethylglyoxime was added. The mixture was allowed to stir for one hour at room temperature and a color change from yellow to red‐orange was observed when 50.0 mg of tromanceniumylboronic acid pinacolato ester triflate (2 equiv, 0.103 mmol) was added. The mixture was allowed to stir for 72 hours and 38.3 μL (9 equiv, 0.459 mmol) of HCl (37 %) was added. After the mixture was stirred for an additional hour, the solvent was removed on a rotary evaporator. The crude product was dissolved with diethyl ether/acetonitrile (7 : 3) and purified by short column chromatography on neutral alumina (1 cm), giving **2** as diamagnetic red‐orange solid in 17.8 % yield (10.4 mg, 0.009 mmol). Mp: >300 °C dec. ^1^H NMR (300 MHz, CD_3_CN, ppm) δ=2.55 (s, 18H, CH_3_), 4.79 (pseudo‐t, 4H of Cp, J=3.0 Hz), 4.84 (pseudo‐t, 4H of Cp, J=3.0 Hz), 6.83 (s, 14H of Cht). ^13^C NMR (75 MHz, CD_3_CN, ppm) δ=14.0 (CH_3_), 79.8 (C2/C5 of Cp), 81.6 (C3/C4 of Cp), 97.6 (C1‐7 of Cht), 154.8 (imine‐carbon). ^11^B NMR (96 MHz, CD_3_CN, ppm) δ=‐11.75. IR (ATR, cm^−1^): 3075, 2925 (ν_C−H_), 1583 (ν_C=N_), 1491, 1446, 1392 (ν_C=C_), 1270, 1253 (ν_B−O_), 1148, 1090 (ν_C−B_), 1028, 999 (ν_N−O_), 634 (ν_Fe_). HRMS (ESI pos, m/z) 420.0644 ([M−OTf]^2+^), calc. for C_36_H_40_B_2_Mn_2_N_6_O_6_Fe: 420.0647. UV‐vis (CH_3_CN, [nm]): λ_max1_=287 (ϵ_1_=9380 L ⋅ mol^−1^ ⋅ cm^−1^), λ_max2_=440 (ϵ_2_=12993 L ⋅ mol^−1^ ⋅ cm^−1^). Anal. calc. for C_38_H_40_B_2_Mn_2_N_6_O_12_FeF_6_S_2_: C 40.10, H 3.54, N 7.38; found: C 40.17, H 3.94, N 6.98. Single crystals were obtained by diffusion crystallization from acetonitrile/diethyl ether at room temperature. **2** is soluble in acetonitrile, ethyl acetate, acetone, and dimethyl sulfoxide, but insoluble in dichloromethane.

**Synthesis of 3**: A round bottom flask was charged with 36.0 mg of CoCl_2_ ⋅ 6H_2_O (1 equiv, 0.151 mmol) and dissolved in 20 mL of methanol. A color change from pink to orange was observed when 52.8 mg (3 equiv, 0.454 mmol) of dimethylglyoxime was added. A color intensification was observed when 100.0 mg of cymantrenylboronic acid pinacolato ester (2 equiv, 0.303 mmol) was added and the mixture was allowed to stir over night. The reaction mixture was filtered through a paper filter and the solvent was removed on a rotary evaporator. The crude product was purified by short column chromatography on silica with diethyl ether/dichloromethane (1 : 1) as eluent affording **3** as paramagnetic orange solid in 26.9 % yield (33.7 mg, 0.041 mmol). Mp: 275 °C dec. IR (ATR, cm^−1^): 2989, 2931 (ν_C−H_), 2014, 1921 (ν_C=O_), 1506 (ν_C=N_), 1491, 1467, 1414 (ν_C=C_), 1272, 1231, 1201, 1168, 1129 (ν_C−B_+ν_B−O_), 900, 848 (ν_N−O_), 662 (ν_Fe_). HRMS (ESI pos, m/z) 828.9870, calc. for C_28_H_26_B_2_Mn_2_N_6_O_12_Co: 828.9888. UV‐vis (CH_2_Cl_2_, [nm]): λ_max1_=279 (ϵ_1_=2536 L ⋅ mol^−1^⋅cm^−1^), λ_max2_=327 (ϵ_2_=2643 L⋅mol^−1^⋅cm^−1^), λ_max3_=466 (ϵ_3_=779 L⋅mol^−1^ ⋅ cm^−1^). Anal. calc. for C_28_H_26_B_2_Mn_2_N_6_O_12_Co: C 40.57, H 3.16, N 10.14; found: C 40.57, H 4.60, N 14.3. Single crystals were obtained by diffusion crystallization from acetone out/pentane at 4 °C. **3** is soluble in diethyl ether, dichloromethane, acetonitrile, acetone, and dimethyl sulfoxide, but insoluble in heptane.

**Synthesis of 4**: A round bottom flask was charged with 36.0 mg of MnCl_2_ ⋅ 4H_2_O (2 equiv, 0.181 mmol) and dissolved in 20 mL of methanol. A color change from pale pink to yellow was observed when 53.0 mg (3 equiv, 0.272 mmol) of 2‐hydroxy‐5‐methylisophthalaldehyde dioxime was added. A color intensification was observed when 60.0 mg of cymantrenylboronic acid pinacolato ester (2 equiv, 0.181 mmol) was added and the mixture was refluxed at 80 °C for 2.5 h. After the reaction mixture has cooled to room temperature, 27.3 μL (3 equiv) of 10 M NaOH was added, whereby the color further intensified from yellow to orange. Additional 60 μL of a 40 % N(Bu)_4_OH solution (1 equiv) was added before the mixture was stirred overnight, while the product partially precipitated. The solvent was removed on a rotary evaporator and purified by column chromatography on silica with dichloromethane/diethyl ether (1 : 1) as eluent. The product was eluted with dichloromethane and concentrated in vacuo to a few mL before it was precipitated with heptane and filtered off, giving **4** as paramagnetic yellow solid in 46.3 % yield (57.0 mg, 0.042 mmol). Mp: 256 °C dec. IR (ATR, cm^−1^): 2963, 2931, 2874 (ν_C−H_), 2005, 1896 (ν_C=O_), 1606 (ν_C=N_), 1579, 1544, 1479 (ν_C=C_), 1441 (δ_as_CH_3_), 1377 (δ_s_CH_3_), 1323, 1223, 1178 (ν_C−B_+ν_B−O_), 636 (ν_Mn_). HRMS (ESI neg, m/z) 1110.9408 ([M−N(Bu)_4_]^−^), calc. for C_43_H_29_B_2_Mn_4_N_6_O_15_: 1110.9404. UV‐vis (CH_3_CN, [nm]): λ_max1_=297 (ϵ_1_=10581 L ⋅ mol^−1^ ⋅ cm^−1^), λ_max2_=360 (ϵ_2_=11621 L ⋅ mol^−1^ ⋅ cm^−1^), λ_max3_=375 (ϵ_2_=11052 L ⋅ mol^−1^ ⋅ cm^−1^). Anal. calc. for C_59_H_65_B_2_Mn_4_N_7_O_15_: C 52.35, H 4.84, N 7.24; found: C 52.69, H 5.03, N 7.25. Single crystals were obtained by diffusion crystallization from acetonitrile/diethyl ether at room temperature. **4** is soluble in dichloromethane, acetonitrile, acetone, dimethyl sulfoxide and insoluble in heptane and methanol.

**Synthesis of 5**: A round bottom flask was charged with 42.8 mg of Co(OAc)_2_ ⋅ 4H_2_O (2 equiv, 0.172 mmol) and dissolved in 20 mL of methanol. A color change from pink to orange was observed when 50.0 mg (3 equiv, 0.257 mmol) of 2‐hydroxy‐5‐methylisophthalaldehyde dioxime was added. After 56.7 mg of cymantrenylboronic acid pinacolato ester (2 equiv, 0.172 mmol) was added, the mixture was refluxed for two hours at 80 °C. The suspension was cooled to room temperature and 25.7 μL (3 equiv) of 10 M NaOH was added. Additional 56.3 μL of a 40 % N(Bu)_4_OH solution (1 equiv) was added before the mixture was stirred for 60 h, while the product partially precipitated. The solvent was removed on a rotary evaporator and purified by column chromatography on silica (3 cm) with dichloromethane. The product was eluted with dichloromethane/acetonitrile (1 : 1). The solvents were removed in vacuo giving **5** as paramagnetic orange solid in 43.2 % yield (50.6 mg, 0.037 mmol). Mp: 208 °C dec. IR (ATR, cm^−1^): 2960, 2928, 2874 (ν_C−H_), 2006, 1889 (ν_C=O_), 1610 (ν_C=N_), 1584, 1552 (ν_C=C_), 1446 (δ_as_CH_3_), 1377 (δ_s_CH_3_), 1322, 1225, 1182 (ν_C−B_+ν_B−O_), 634 (ν_Co_). HRMS (ESI neg, m/z) 1118.9320 ([M−N(Bu)_4_]^−^), calc. for C_43_H_29_B_2_Mn_2_Co_2_N_6_O_15_: 1118.9307. UV‐vis (CH_3_CN, [nm]): λ_max1_=296 (ϵ_1_=11294 L ⋅ mol^−1^ ⋅ cm^−1^), λ_max2_=376 (ϵ_2_=11174 L ⋅ mol^−1^ ⋅ cm^−1^). Anal. calc. for C_59_H_65_B_2_Co_2_Mn_2_N_7_O_15_: C 52.05, H 4.81, N 7.20; found: C 52.26, H 5.02, N 6.67. Single crystals were obtained by diffusion crystallization from acetone/pentane at room temperature. **5** is soluble in dimethyl ether, dichloromethane, acetonitrile, acetone and dimethyl sulfoxide.

**Synthesis of 6**: A round bottom flask was charged with 20.5 mg of Co(OAc)_2_ ⋅ 4H_2_O (2 equiv, 0.082 mmol) and dissolved in 20 mL of methanol. A color change from a pink solution to an orange suspension was observed when 24.0 mg (3 equiv, 0.123 mmol) of 2‐hydroxy‐5‐methylisophthalaldehyde dioxime was added. After 40.0 mg of tromanceniumylboronic acid pinacolato ester triflate (2 equiv, 0.041 mmol) was added, the mixture was refluxed for twenty‐four hours at 80 °C. The orange suspension was cooled to room temperature before 12.3 μL (3 equiv) of 10 M NaOH was added, while the orange precipitate dissolved, accompanied by a color intensification. The solvent was removed on a rotary evaporator giving a brown solid, which was then washed with diethyl ether and dissolved again with acetonitrile. Insoluble remaining was filtered off through a folded filter. The solvent was removed in vacuo giving pure **6** as a paramagnetic orange solid in 85.5 % yield (45.1 mg, 0,035 mmol). Mp: 250 °C dec. IR (ATR, cm^−1^): 3077, 3005, 2927, 2856 (ν_C−H_), 1611, 1557 (ν_C=N_+ν_C=C_), 1447 (δ_as_CH_3_), 1382 (δ_s_CH_3_), 1323, 1255, 1228, 1169 (ν_C−B_+ν_B−O_), 1004, 961 (ν_N−O_), 634 (ν_Mn_). HRMS (ESI pos, m/z) 1133.0702 ([M−OTf]^+^), calc. for C_51_H_43_B_2_Mn_2_Co_2_N_6_O_9_: 1133.0697. UV‐vis (CH_3_CN, [nm]): λ_max1_=295 (ϵ_1_=10683 L ⋅ mol^−1^ ⋅ cm^−1^), λ_max2_=376 (ϵ_2_=8339 L ⋅ mol^−1^ ⋅ cm^−1^). Combustion analysis was attempted, but despite repeated attempts no reasonable data could be obtained. **6** is soluble in acetonitrile, acetone, nitromethane and dimethyl sulfoxide.

**Synthesis of 7**: A round bottom flask was charged with 20.5 mg of MnCl_2_ ⋅ 4H_2_O (2 equiv, 0.156 mmol) and dissolved in 20 mL of methanol. A color change from colorless to yellow was observed when 45.7 mg (3 equiv, 0.235 mmol) of 2‐hydroxy‐5‐methylisophthalaldehyde dioxime was added. A second color change from yellow to cherry red was observed when 76.2 mg of tromanceniumylboronic acid pinacolato ester triflate (2 equiv, 0.156 mmol) was added. The mixture was refluxed for one and a half hours at 80 °C. The red solution was cooled to room temperature before 47.0 μL (6 equiv) of 10 M NaOH was added, while a skin‐colored solid precipitated. The suspension was refluxed overnight, accompanied by a color intensification. The solvent was removed on a rotary evaporator giving a brown solid, which was then washed with diethyl ether and dissolved again with acetonitrile. Insoluble remaining was filtered off through a paper filter. The solvent was removed in vacuo giving pure **7** as a paramagnetic beige solid in 15.2 % yield (15.1 mg, 0,012 mmol). Mp: >300 °C dec. IR (ATR, cm^−1^): 3075, 2971, 2943, 2919, 2853 (ν_C−H_), 1608, 1580, 1546 (ν_C=N_+ν_C=C_), 1439 (δ_as_CH_3_), 1383 (δ_s_CH_3_), 1321, 1260, 1223, 1195, 1173 (ν_C−B_+ν_B−O_), 993, 938 (ν_N−O_), 647 (ν_Mn_). HRMS (ESI pos, m/z) 1125.0784 ([M−OTf]^+^), calc. for C_51_H_43_B_2_Mn_4_N_6_O_9_: 1125.0794. UV‐vis (CH_3_CN, [nm]): λ_max1_=297 (ϵ_1_=10748 L ⋅ mol^−1^ ⋅ cm^−1^), λ_max2_=360 (ϵ_2_=10928 L ⋅ mol^−1^ ⋅ cm^−1^). Combustion analysis was attempted, but despite repeated attempts no reasonable data could be obtained. **7** is soluble in dichloromethane, acetonitrile, acetone and dimethyl sulfoxide.

**Cytotoxicity studies**: Cell lines were obtained from DSMZ‐German Collection of Microorganisms and Cell Cultures. The cytotoxic effects were determined according to standard protocols. In short: A volume of 100 μL of HT‐29 cells (15,500 cells/mL), A549 cells (9,800 cells/mL), MCF‐7 cells (5,300 cells/mL) or VeroE6 (5,700 cells/mL) was transferred into the wells of a 96‐well plates and incubated at 37 °C under 5 % CO_2_ for 72 h. Stock solutions of the compounds were freshly prepared in dimethyl sulfoxide (DMSO) and diluted with the cell culture medium to obtain various concentrations (final concentration of DMSO: 0.1 % v/v). After 72 h (HT‐29, A549) or 96 h (MCF‐7, VeroE6) of exposure, the biomass of the cells was determined via crystal violet staining and the IC_50_ value was determined as the concentration that caused 50 % inhibition of cell proliferation relative to an untreated control. The results were calculated as the mean values of two independent experiments.

## Supporting Information

Spectra (^1^H/^11^B/^13^C NMR, IR, UV‐vis, HRMS) and cyclic voltammograms of complexes **1**–**7**.

Deposition Numbers 2253707 (for **1**), 2253708 (for **2**), 2253709 (for **3**), 2253710 (for **4**), and 2253711 (for **5**) contain the supplementary crystallographic data for this paper. These data are provided free of charge by the joint Cambridge Crystallographic Data Centre and Fachinformationszentrum Karlsruhe Access Structures service.

## Conflict of interest

The authors declare no conflict of interest.

1

## Supporting information

As a service to our authors and readers, this journal provides supporting information supplied by the authors. Such materials are peer reviewed and may be re‐organized for online delivery, but are not copy‐edited or typeset. Technical support issues arising from supporting information (other than missing files) should be addressed to the authors.

Supporting Information

## Data Availability

The data that support the findings of this study are available in the supplementary material of this article.

## References

[1] Monographs and reviews:

[1a] Y. Z. Voloshin , L. G. Belaya , R. K. Krämer , Cage Metal Complexes; Clathrochelates Revisited, Springer International Publishing: New York, 2017;

[1b] Y. Z. Voloshin , N. A. Kostramina , R. K. Krämer , Clathrochelates: Synthesis, Structure and Properties, Elsevier Science: Amsterdam, The Netherlands, 2002;

[1c] S. M. Jansze , K. Severin , Acc. Chem. Res. 2018, 51, 2139–2147. 30156828 10.1021/acs.accounts.8b00306

[2] Y. Z. Voloshin , V. M. Buznik , A. G. Dedov , Pure Appl. Chem. 2020, 92, 1159–1174.

[3a] Y. Z. Voloshin , V. V. Novikov , Y. V. Nelyubina , RSC Adv. 2015, 5, 72621–72637;

[3b] O. A. Varzatskii , A. V. Vologzhanina , V. V. Novikov , S. Vakarov , R. V. Oblap , Y. Z. Voloshin , Inorg. Chim. Acta 2018, 482, 90–98.

[4a] R. Basse , S. Vanicek , T. Höfer , H. Kopacka , K. Wurst , T. Müller , H. A. Schwartz , S. Olthof , L. A. Casper , M. Nau , R. F. Winter , M. Podewitz , B. Bildstein , Organometallics 2021, 40, 2736–2749; 34393320 10.1021/acs.organomet.1c00376 PMC8356223

[4b] R. Thaler , H. Kopacka , K. Wurst , T. Müller , D. F. Dinu , K. R. Liedl , F. R. Neururer , S. Hohloch , B. Bildstein , Organometallics 2022, 41, 1464–1473; 36157257 10.1021/acs.organomet.2c00179 PMC9490842

[4c] A. Pavun , H. Kopacka , K. Wurst , T. Müller , F. R. Neururer , S. Hohloch , B. Bildstein , J. Organomet. Chem. 2023, 985, 122594;

[4d] R. Thaler , K. Wurst , B. Bildstein , IUCrData 2023, 8, x230107. 36911078 10.1107/S2414314623001074 PMC9993894

[5] S. Khanra , T. Weyhermüller , E. Bill , P. Chaudhuri , Inorg. Chem. 2006, 45, 5911–5923. 16841996 10.1021/ic060409a

[6a] D. F. Evans, *J. Chem. Soc*. **1959**, 2003–2005;

[6b] D. F. Evans, G. V. Fazakerley, R. F. Phillips, *J. Chem. Soc. A* **1971**, 1931–1934.

[7a] A. J. Campanella , T. M. Ozvat , J. M. Zadrozny , Dalton Trans. 2022, 51, 3341–3348; 35137732 10.1039/d1dt02156g PMC8992015

[7b] A. S. Belov , S. A. Belova , N. N. Efimov , V. V. Zlobina , V. V. Novikov , Y. V. Nelyubina , Y. V. Zubavichus , Y. Z. Voloshin , A. A. Pavlov , Dalton Trans. 2023, 52, 2928–2932; 36811361 10.1039/d2dt04073e

[7c] M. Marmier , G. Cecot , A. V. Vologzhanina , J. L. Bila , I. Zivkovic , H. M. Ronnow , B. Nafradi , E. Solari , P. Pattison , R. Scopelliti , K. Severin , Dalton Trans. 2016, 45, 15507–15516. 27722643 10.1039/c6dt02758j

[8a] Y. Z. Voloshin , O. Varzatskii , A. S. Belov , Z. A. Starikova , A. V. Dolganov , V. V. Novikov , Y. N. Bubnov , Inorg. Chim. Acta 2011, 370, 322–332;

[8b] M. Pascu , M. Marmier , C. Schouwey , R. Scopelliti , J. J. Holstein , G. Bricogne , K. Severin , Chem. Eur. J. 2014, 20, 5592–5600; 24700760 10.1002/chem.201400285

[8c] C. Ge , J. Zhang , Z. Qin , P. Zhang , R. Zhang , H. Zhao , Y. Wang , X. Zhang , Inorg. Chim. Acta 2017, 463, 134–141;

[8d] O. M. Planes , R. Scopelliti , F. Fadaei-Tirani , K. Severin , Z. Anorg. Allg. Chem. 2021, 647, 1065–1069.

[9a] S. Sen , M. Won , M. S. Levine , Y. Noh , A. C. Sedgwick , J. S. Kim , J. L. Sessler , J. F. Arambula , Chem. Soc. Rev. 2022, 51, 1212–1233; 35099487 10.1039/d1cs00417d PMC9398513

[9b] E. J. Anthony , E. M. Bolitho , H. E. Bridgewater , O. W. Carter , J. M. Donelly , C. Imberti , E. C. Lant , F. Lermyte , R. J. Needham , M. Palau , P. J. Sadler , H. Shi , F.-X. Wang , W.-Y. Zhang , Z. Zhang , Chem. Sci. 2020, 11, 12888–12917; 34123239 10.1039/d0sc04082g PMC8163330

[9c] P. Zhang, P. J. Sadler, *Eur. J. Inorg. Chem*. **2017**, 1541–1548;

[9d] K. Peng , Y. Zheng , W. Xia , Z.-W. Mao , Chem. Soc. Rev. 2023, 52, 2790–2832. 37014670 10.1039/d2cs00757f

[10] G. Moreno-Alcantar , A. Casini , FEBS Lett. 2023, 597, 191–202. 36345593 10.1002/1873-3468.14535

