# Supplementary material for: Clathrochelate Complexes Containing Axial Cymantrene and Tromancenium Moieties
Source: Eur J Inorg Chem. 2023 Jul 17;26(26):e202300368. doi: 10.1002/ejic.202300368 (PMC10947045; doi:10.1002/ejic.202300368)
Supplement: Supplementary file 1 — Supporting Information [file EJIC-26-0-s001.pdf]

European Journal of Inorganic Chemistry

Supporting Information

Clathrochelate Complexes Containing Axial Cymantrene and Tromancenium Moieties

Reinhard Thaler, Holger Kopacka, Klaus Wurst, Thomas Müller, Florian R. Neururer, Stephan Hohloch, Petra Lippmann, Ingo Ott,* and Benno Bildstein*

1. ANALYTICAL SECTION 1
CLATHROCHELATE (1) 1
CLATHROCHELATE (2) 4
CLATHROCHELATE (3) 7
CLATHROCHELATE (4) 9
CLATHROCHELATE (5) 11
CLATHROCHELATE (6) 13
CLATHROCHELATE (7) 15
2. CYCLIC VOLTAMMETRY 17
CLATHROCHELATE (1) 17
CLATHROCHELATE (2) 17
CLATHROCHELATE (3) 18
CLATHROCHELATE (4) 20
CLATHROCHELATE (5) 21
CLATHROCHELATE (6) 21

1. Analytical Section

Clathrochelate (1)

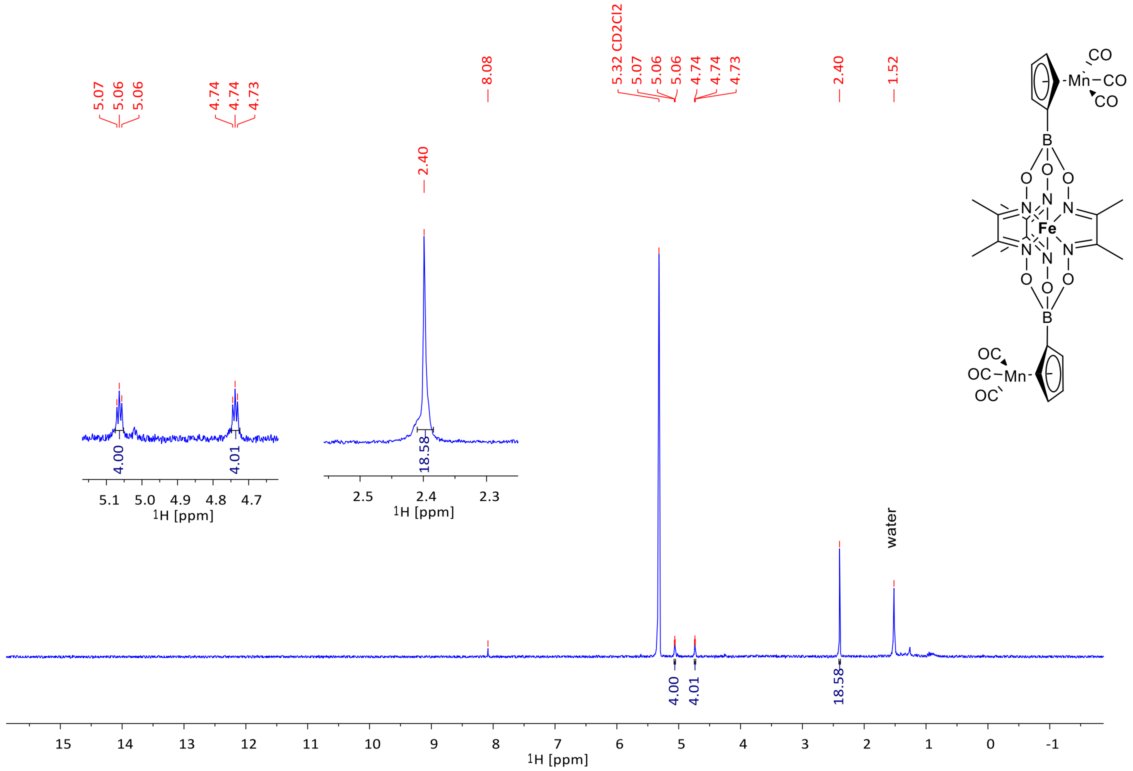

Figure S1. ^{1}H-NMR spectrum of **1**.

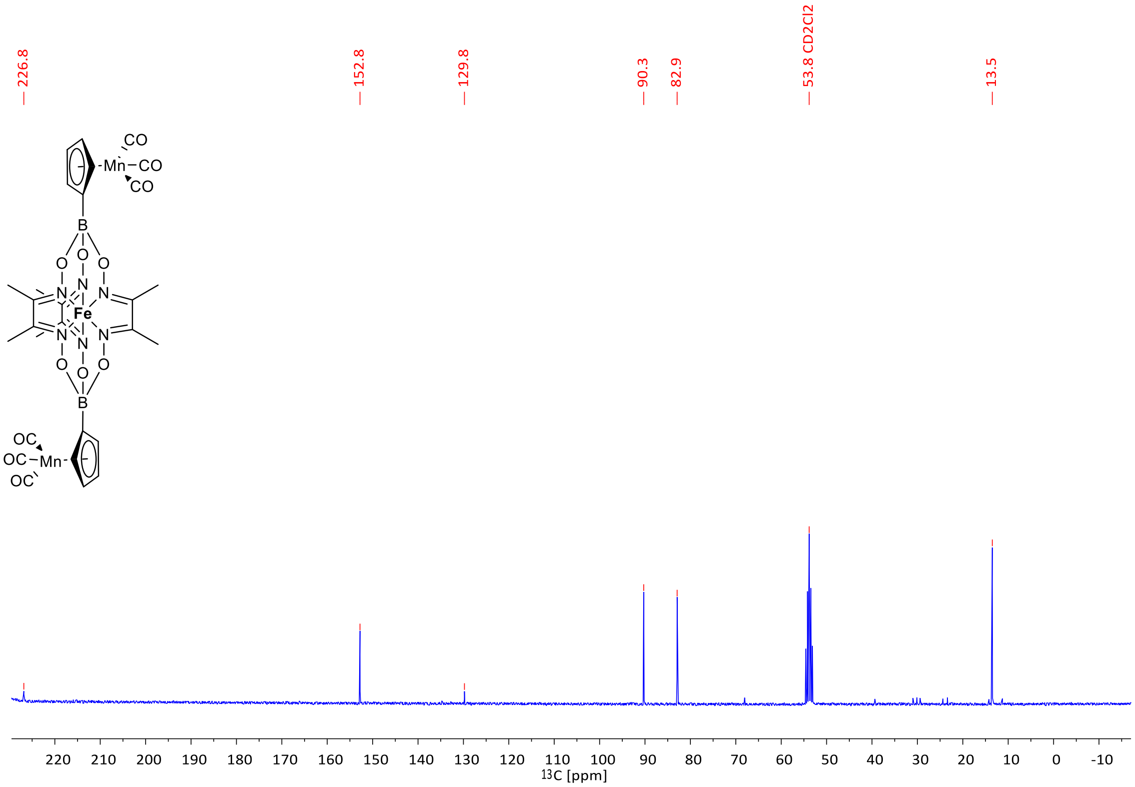

Figure S2. ^{13}C-NMR spectrum of **1**.

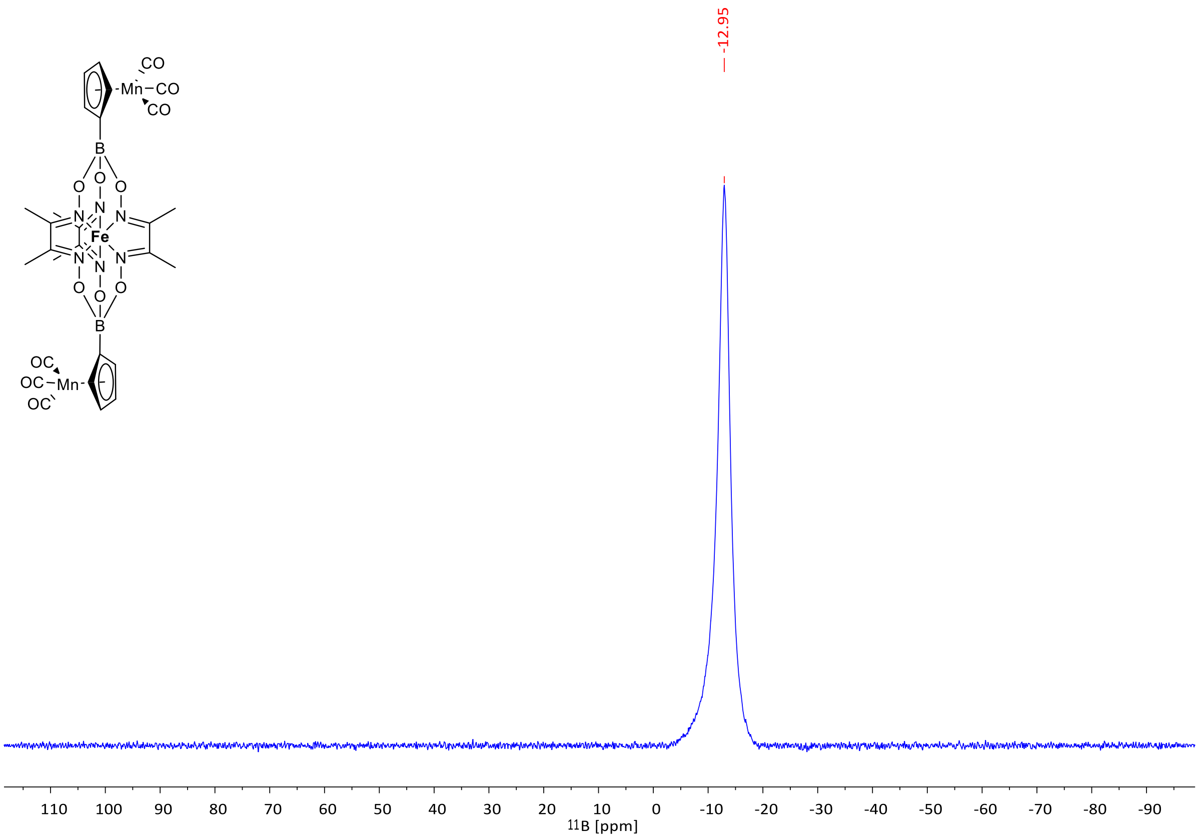

Figure S3. ^{11}B-NMR spectrum of **1**.

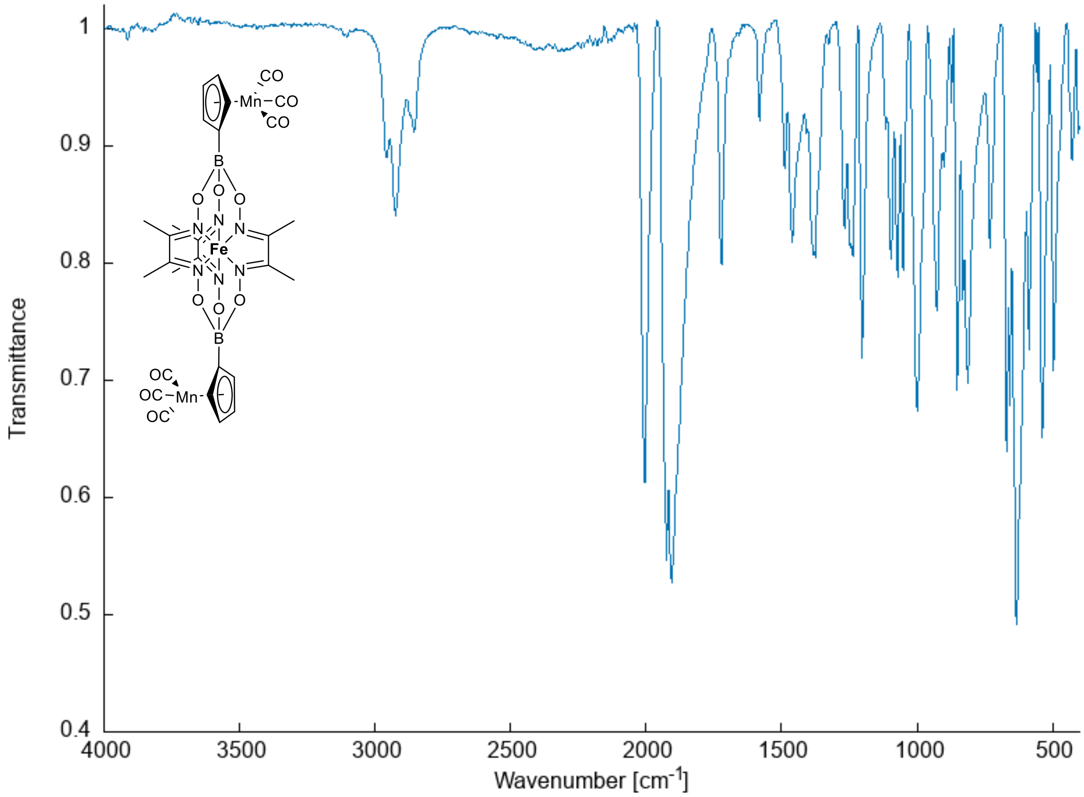

Figure S4. IR spectrum of **1**.

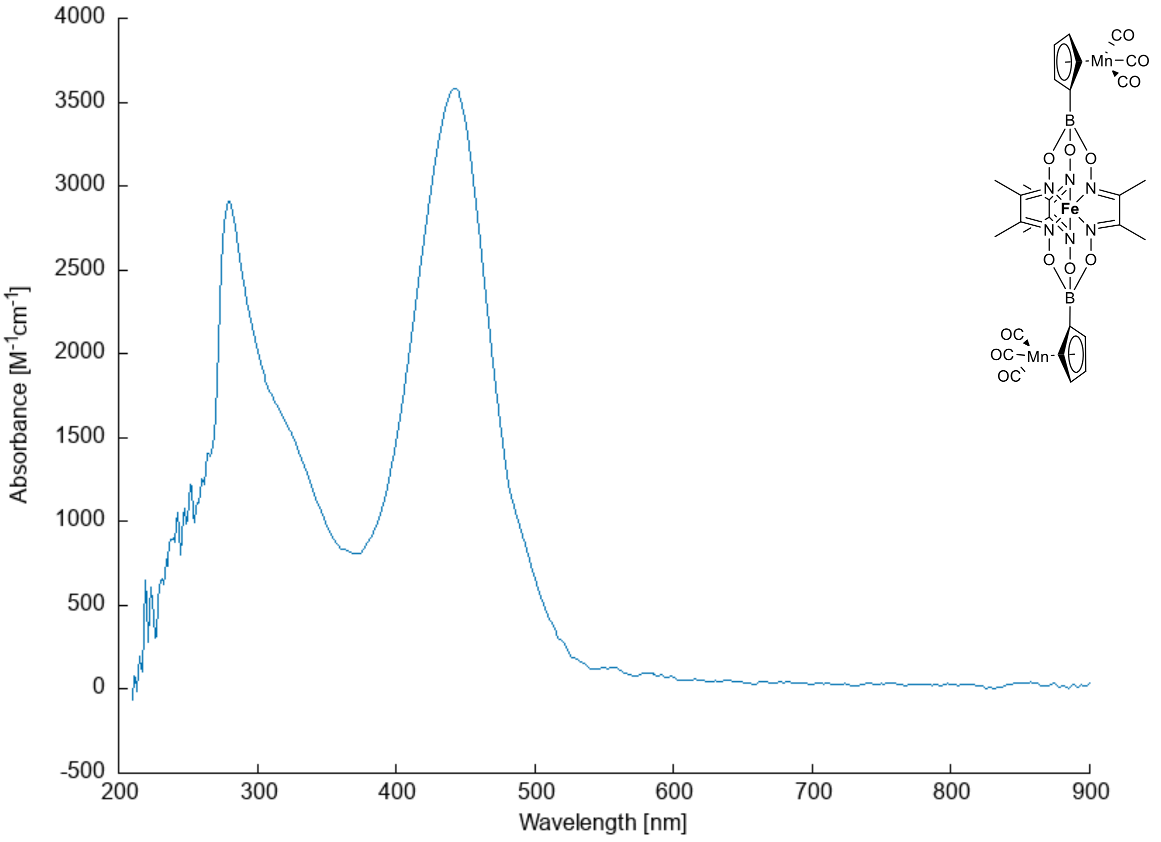

Figure S5. UV/vis spectrum of **1**.

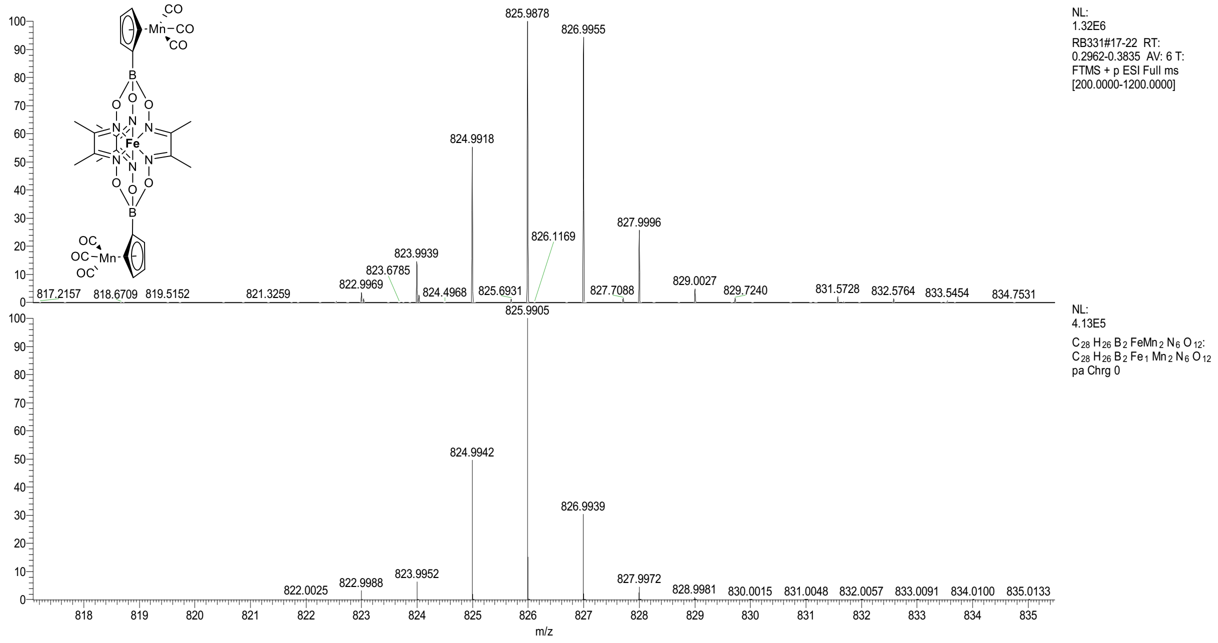

Figure S6. MS (ESI pos, [m/z]; *top*: experimental, *bottom*: simulated) of **1**.

Clathrochelate (2)

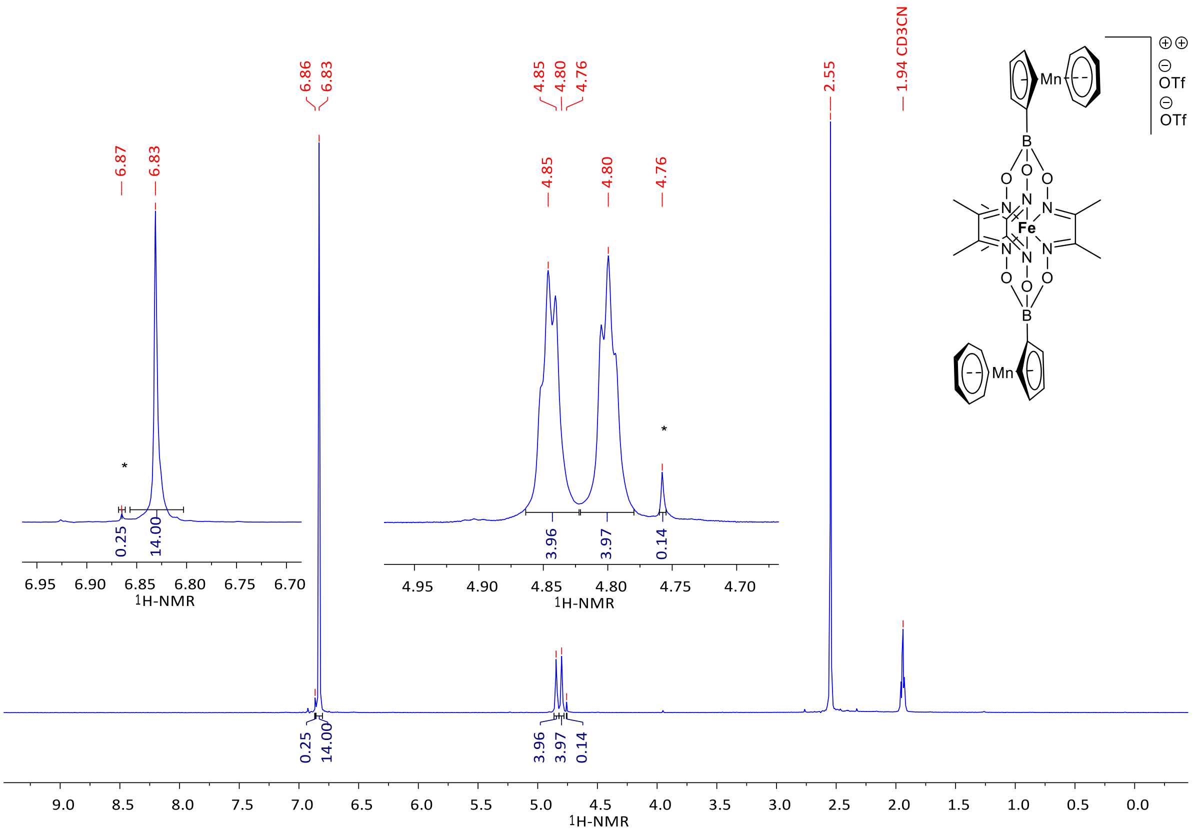

Figure S7. ^{1}H-NMR spectrum of **2** (marked: traces of unsubstituted Tromancenium).

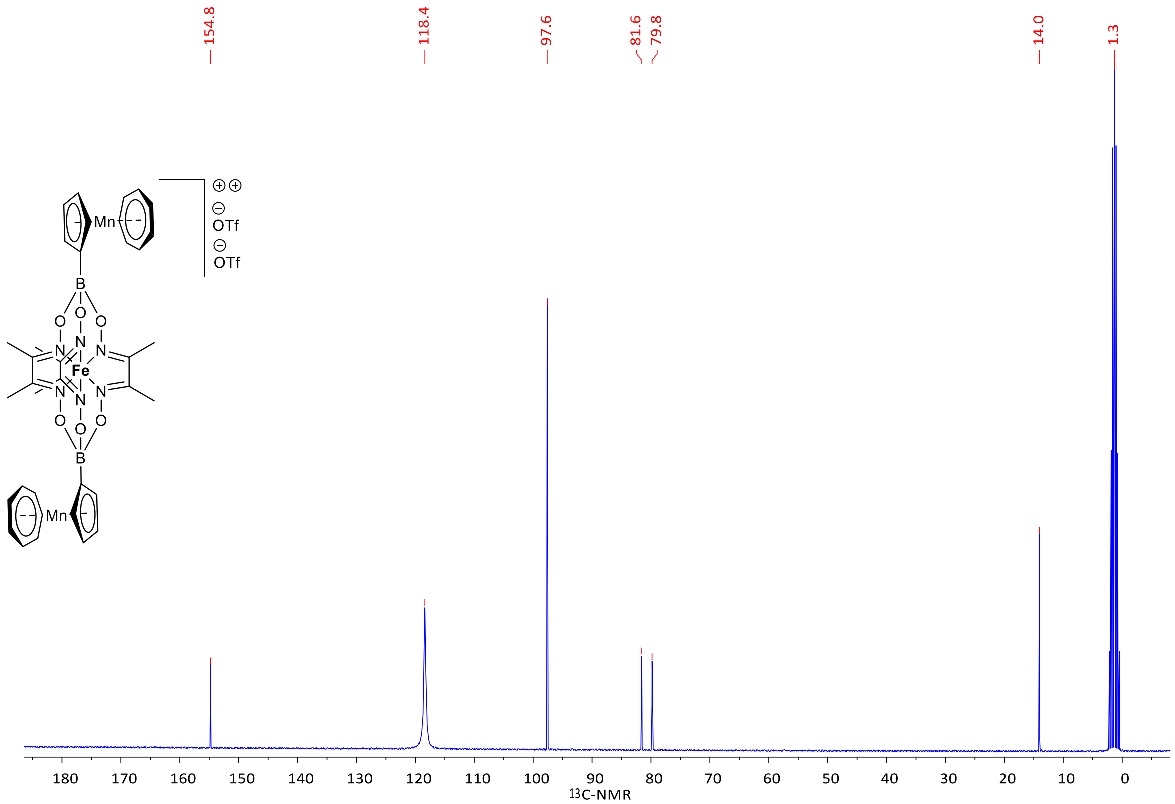

Figure S8. ^{13}C-NMR spectrum of **2**.

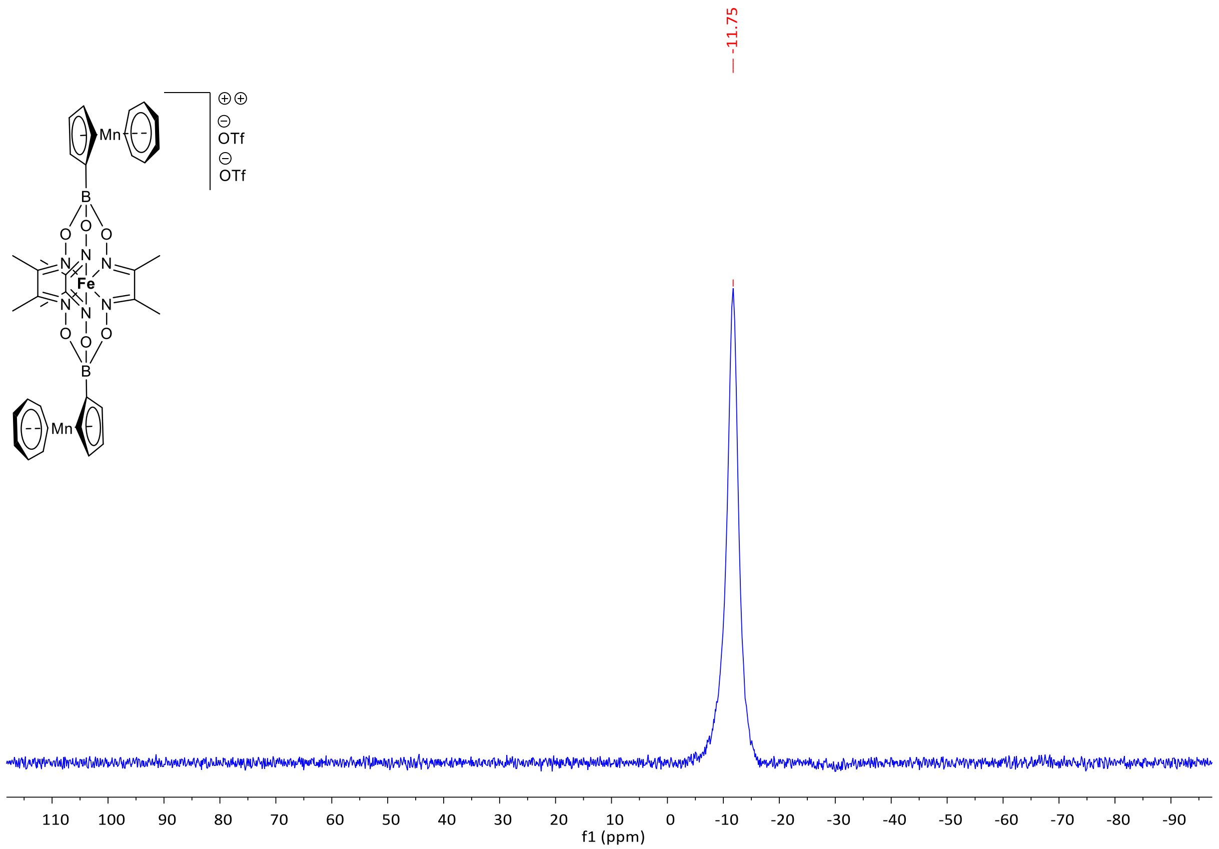

Figure S9. ^{11}B-NMR spectrum of **2**.

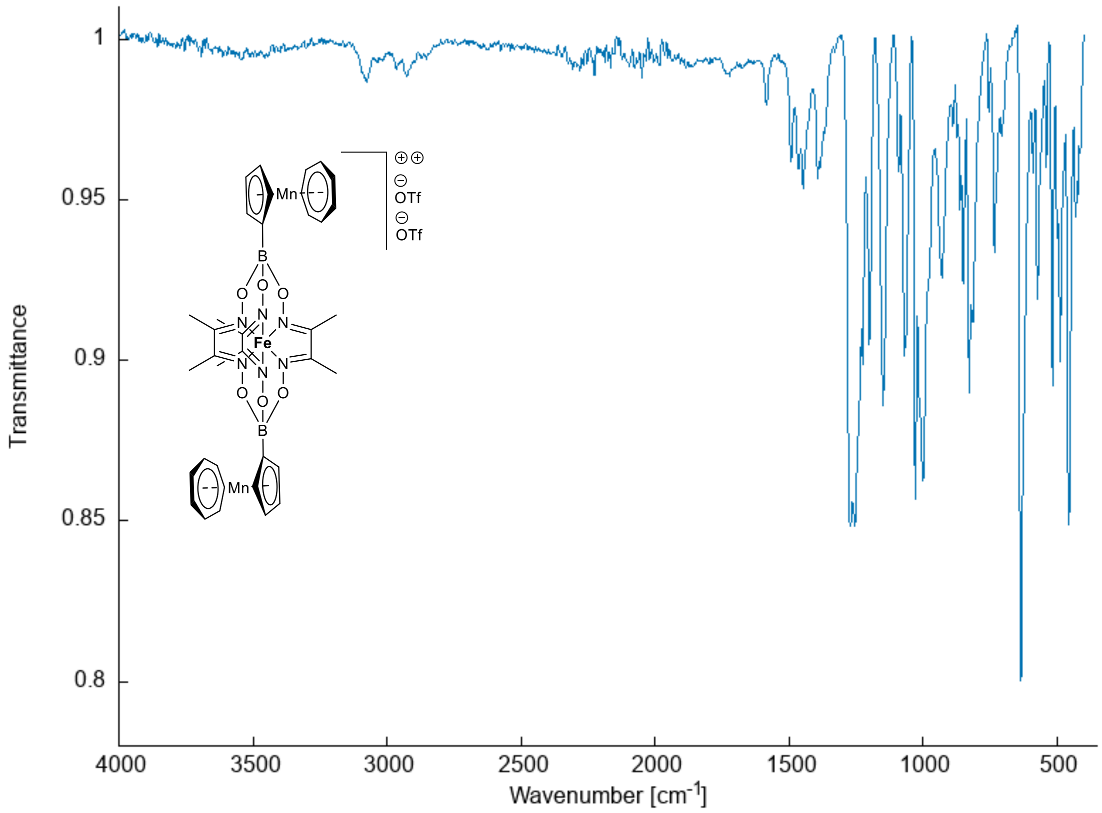

Figure S10. IR spectrum of **2**.

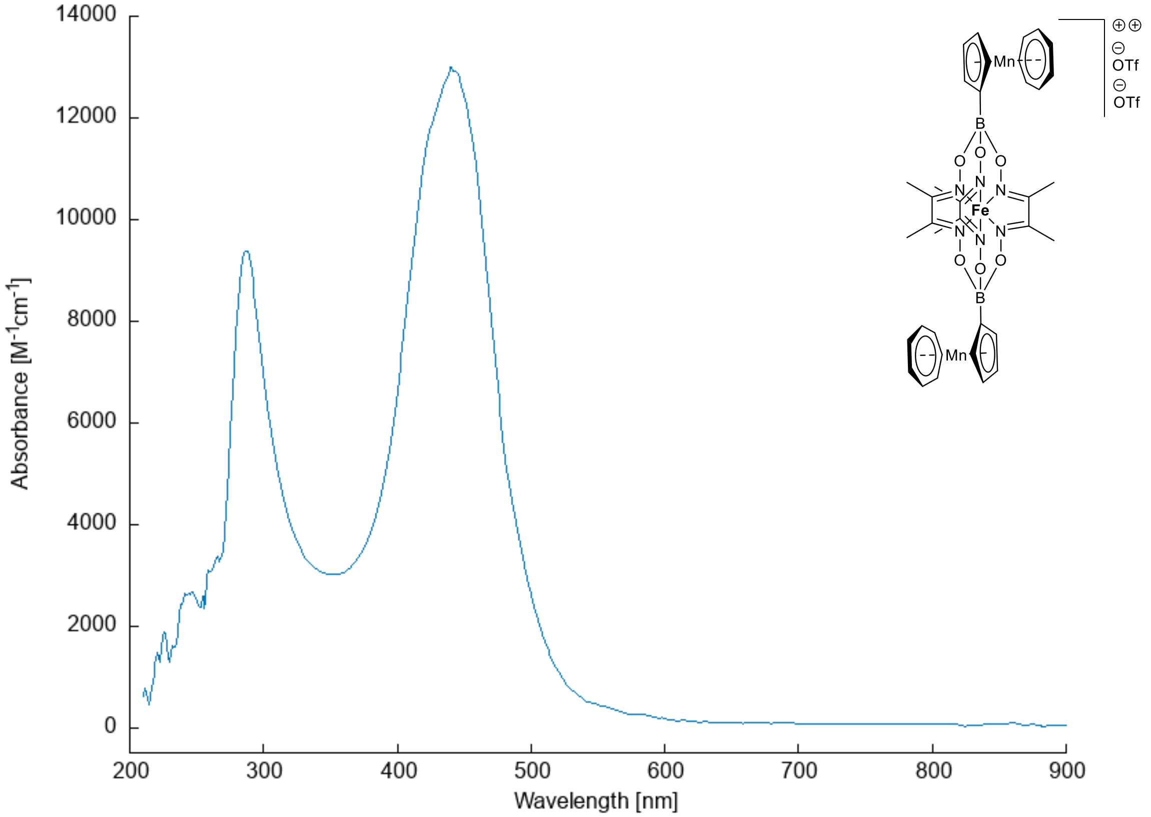

Figure S11. UV/vis spectrum of **2**.

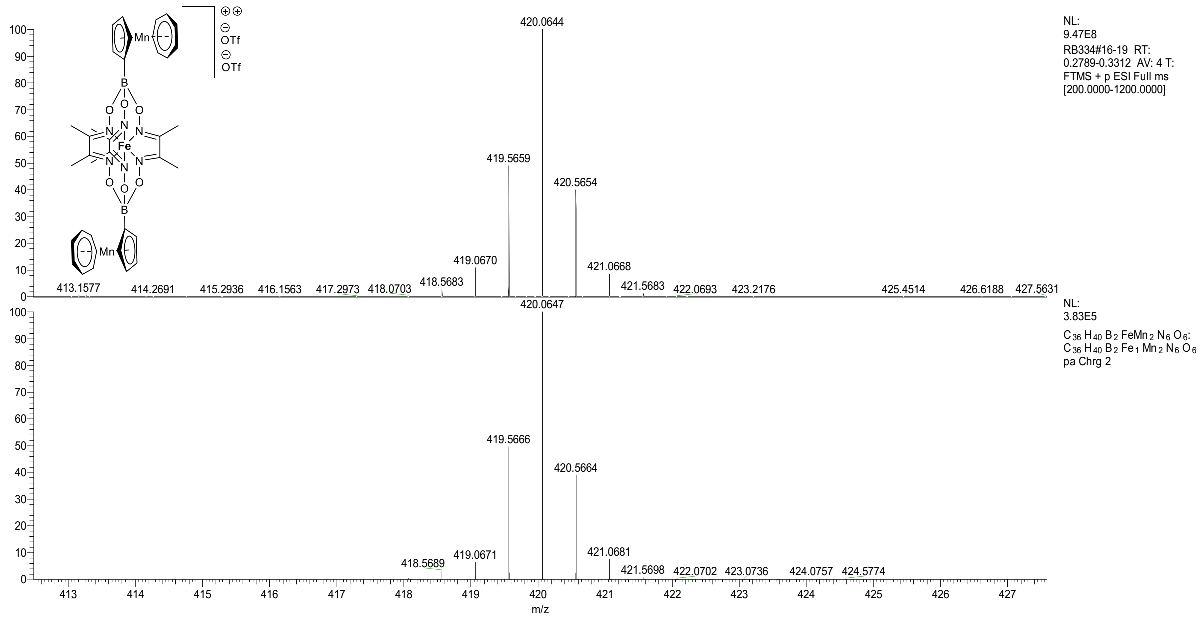

Figure S12. MS (ESI pos, [m/z]; *top*: experimental, *bottom*: simulated) of **2**.

Clathrochelate (3)

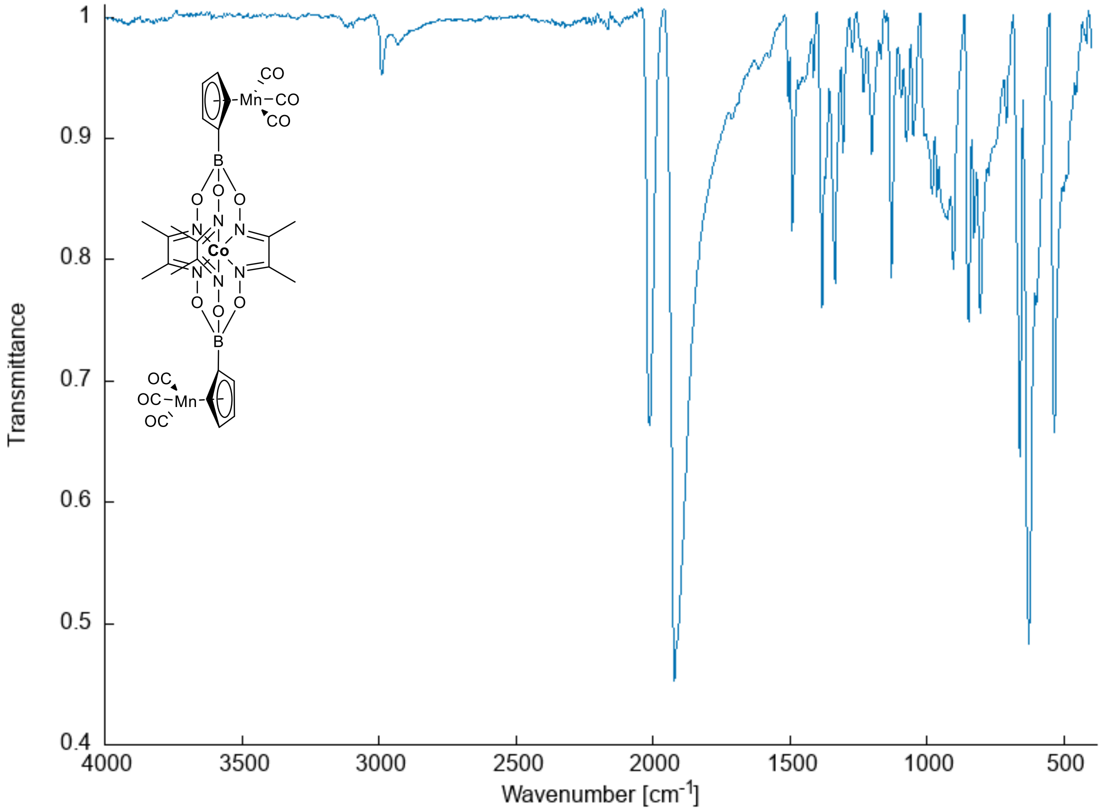

Figure S13. IR spectrum of **3**.

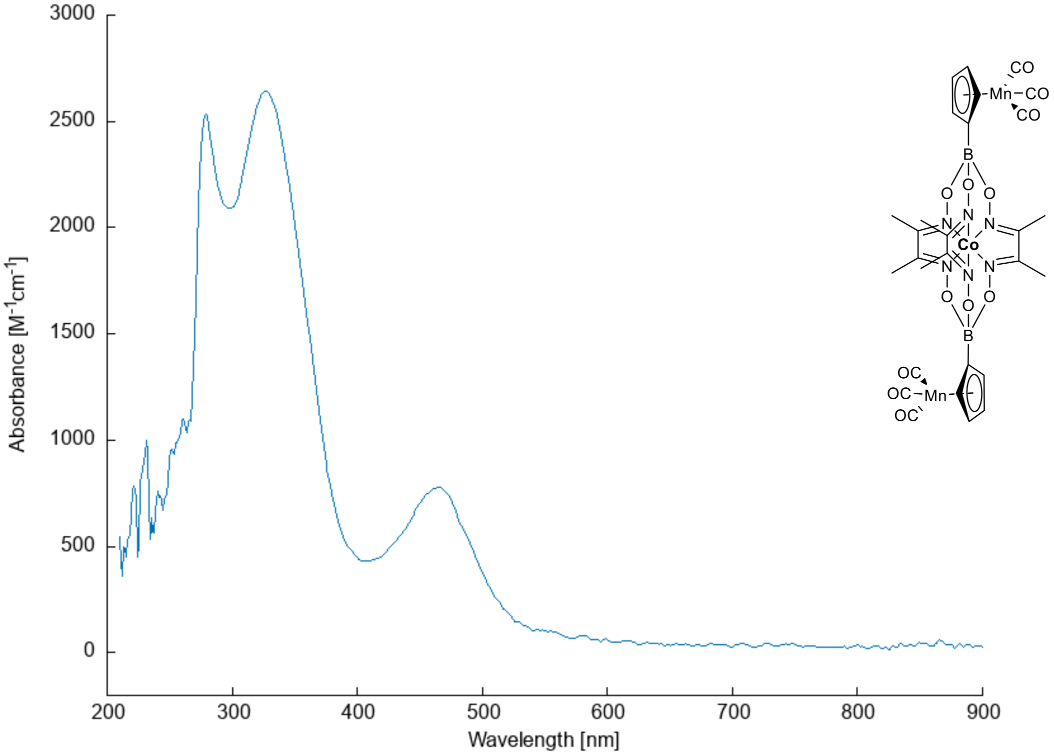

Figure S14. UV/vis spectrum of **3**.

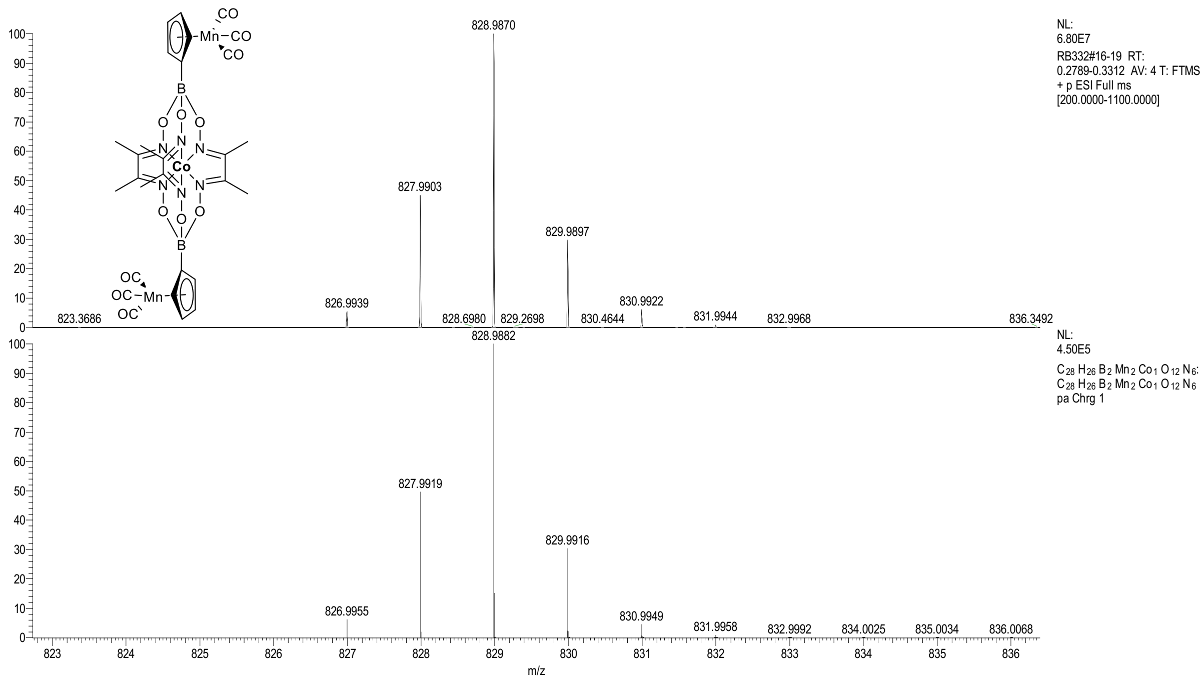

Figure S15. MS (ESI pos, [m/z]; *top*: experimental, *bottom*: simulated) of **3**

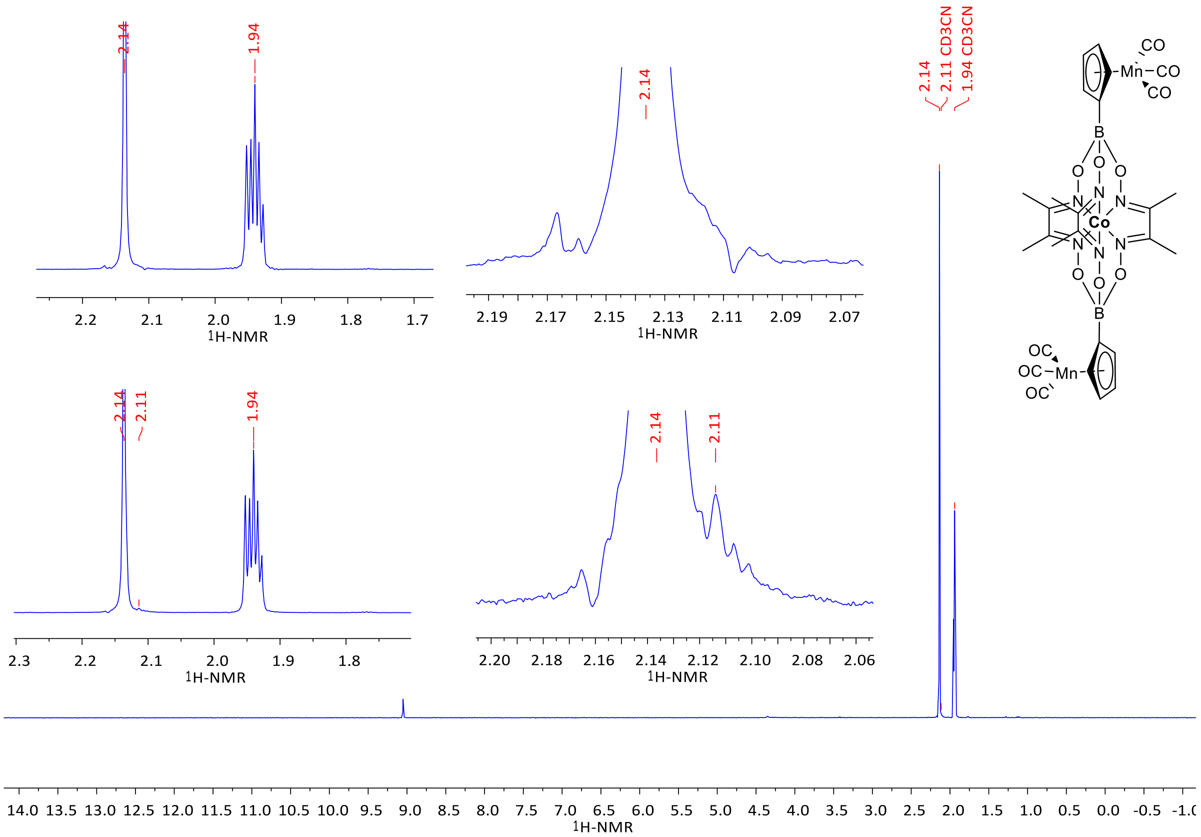

Figure S16. Top: ^{1}H-NMR spectrum of **3**. Bottom: ^{1}H-NMR spectrum of **3** with CD_3CN capillary.

Clathrochelate (4)

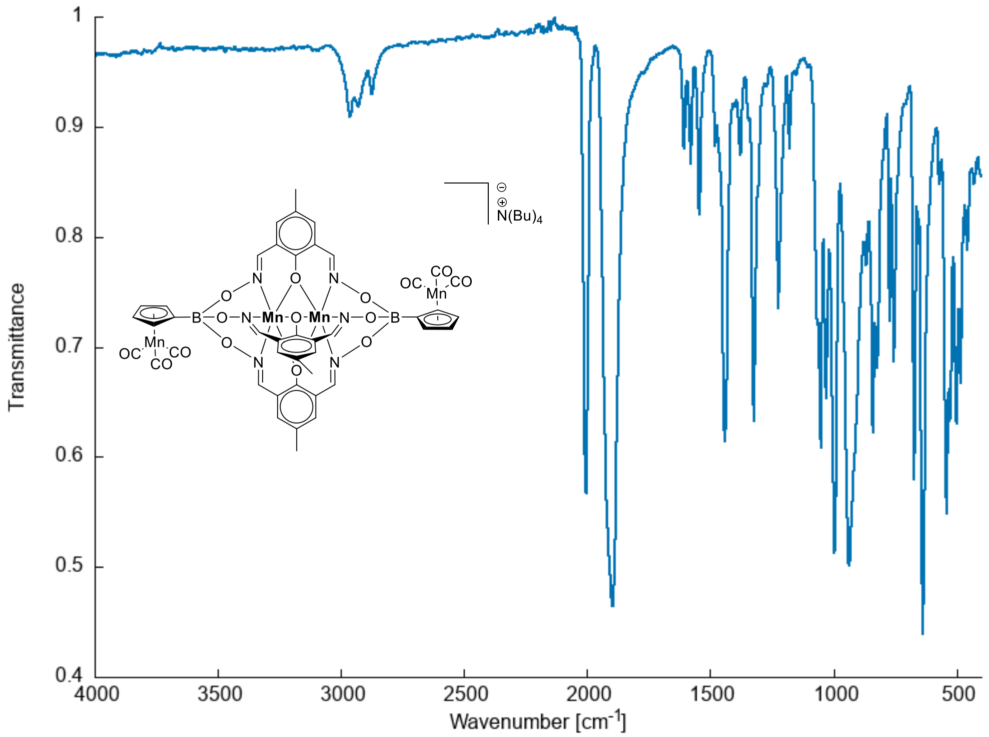

Figure S17. IR spectrum of **4**.

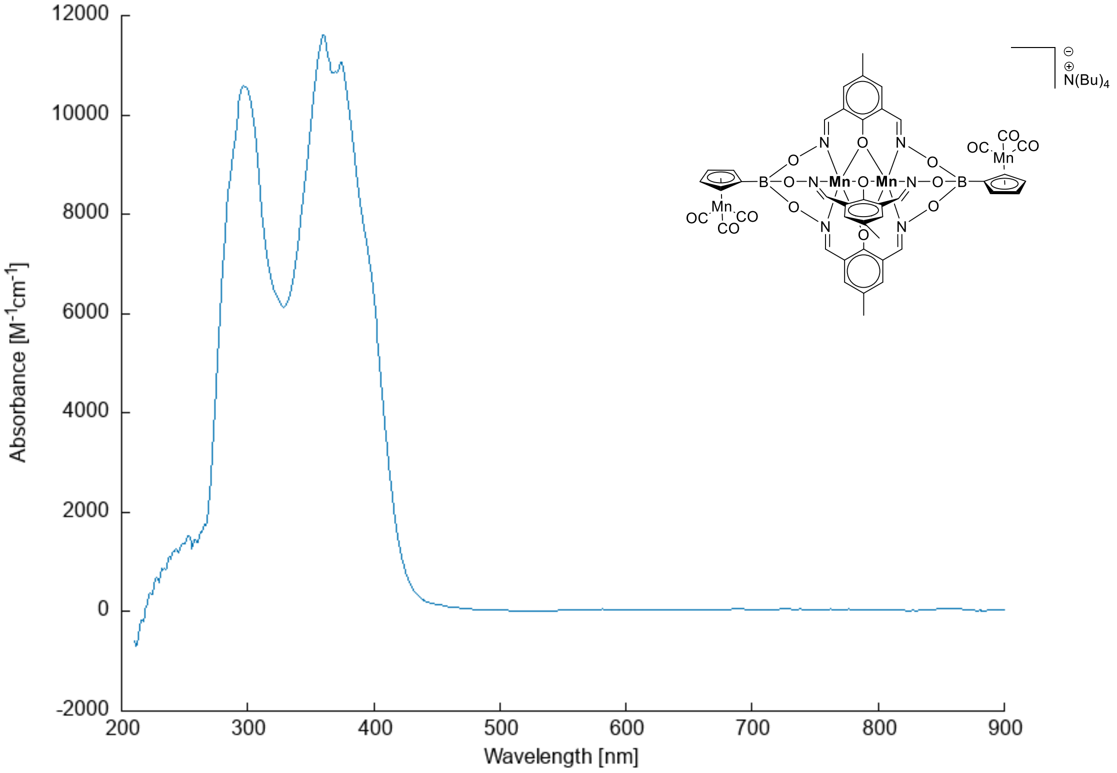

Figure S18. UV/vis spectrum of **4**.

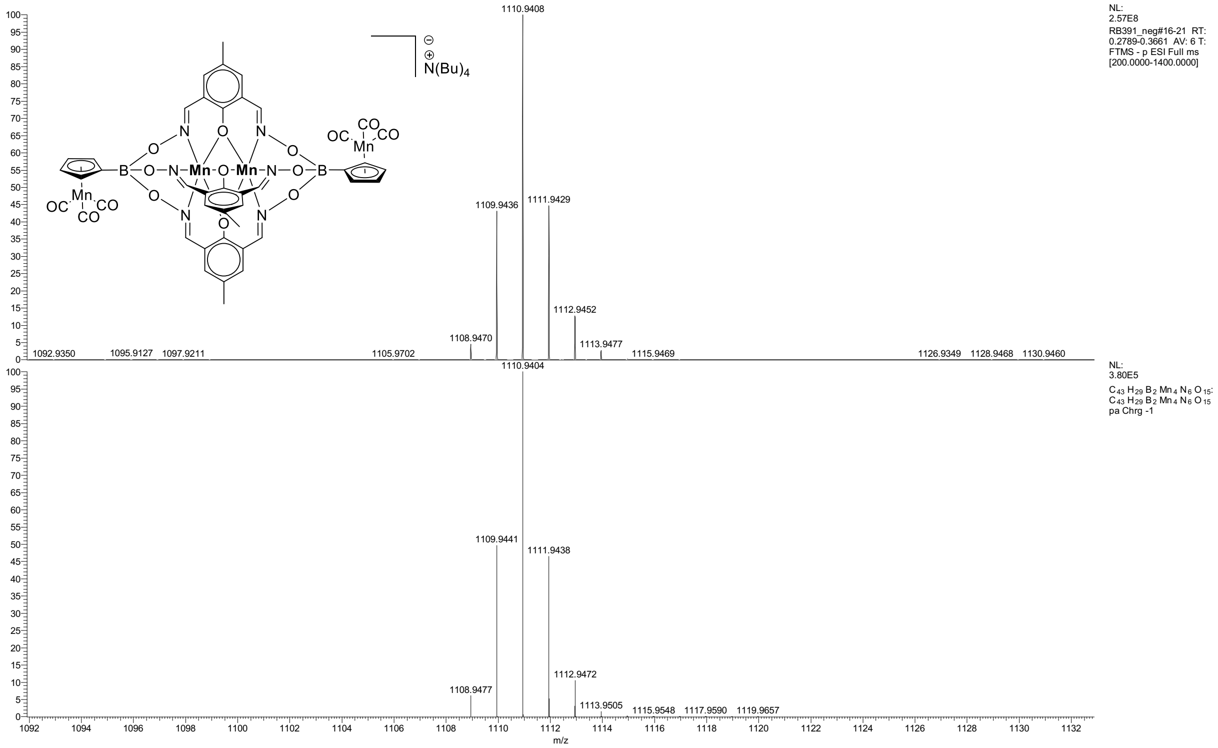

Figure S19. MS (ESI pos, [m/z]; *top*: experimental, *bottom*: simulated) of **4**.

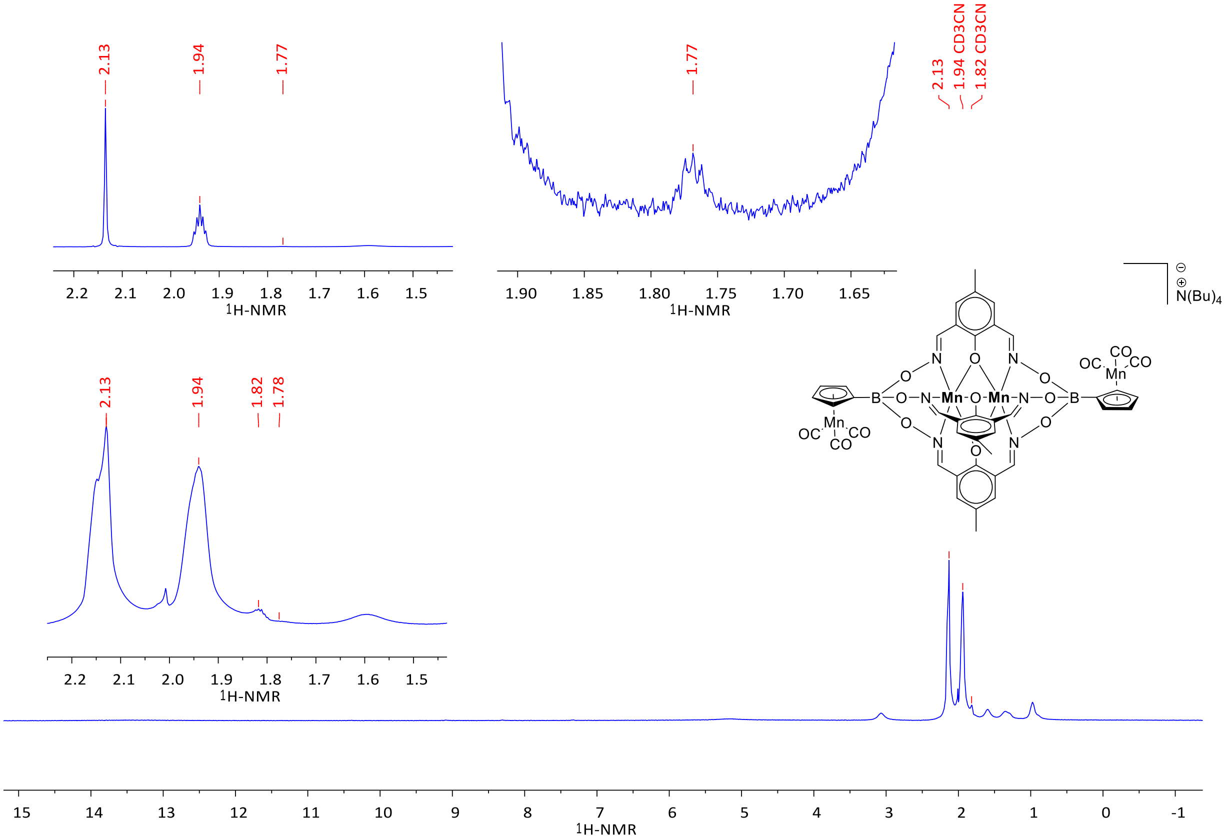

Figure S20. Top: ^{1}H-NMR spectrum of **4**. Bottom: ^{1}H-NMR spectrum of **4** with CD_3CN capillary.

Clathrochelate (5)

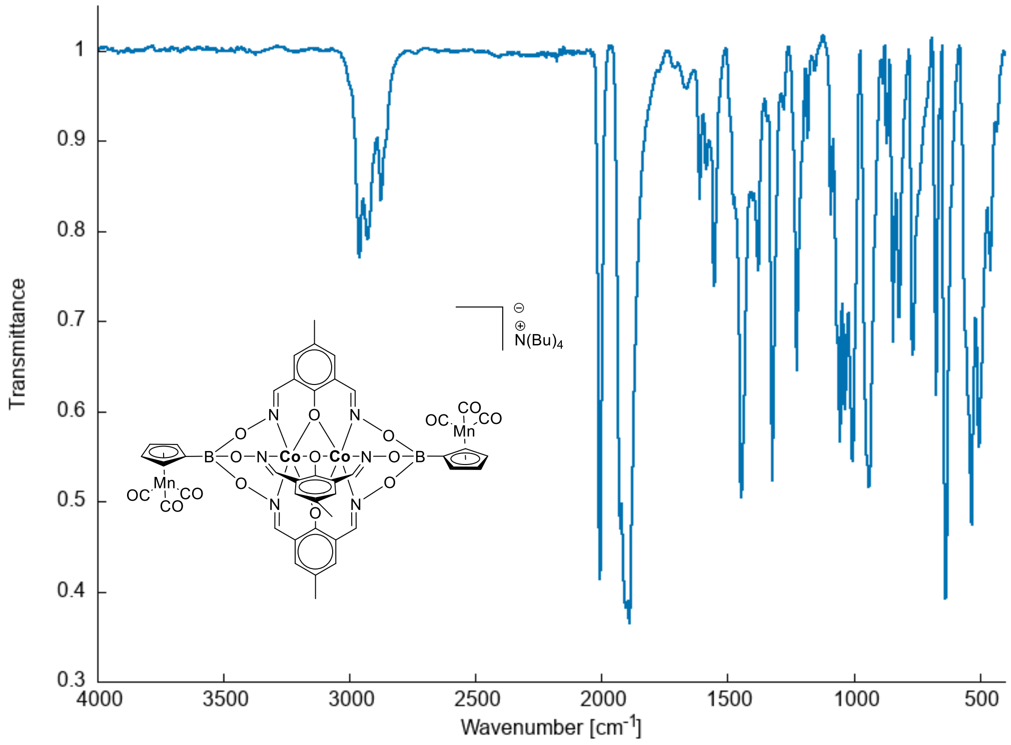

Figure S21. IR spectrum of **5**.

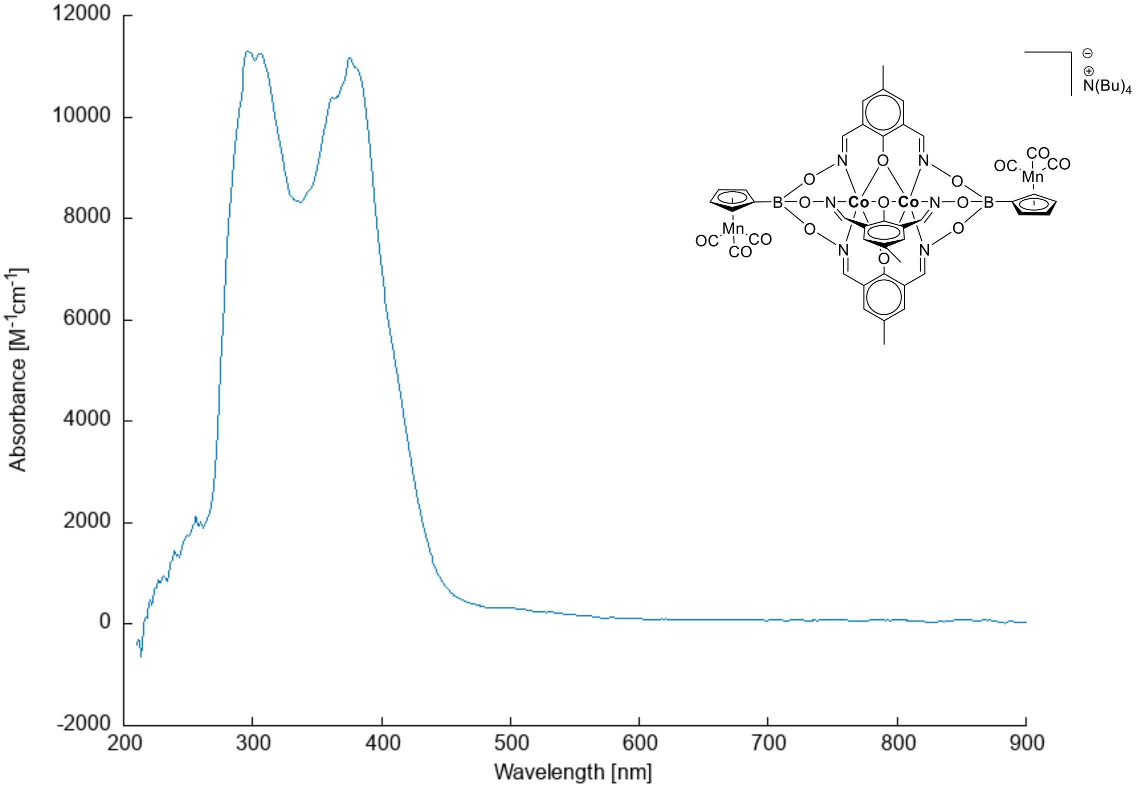

Figure S22. UV/vis spectrum of **5**.

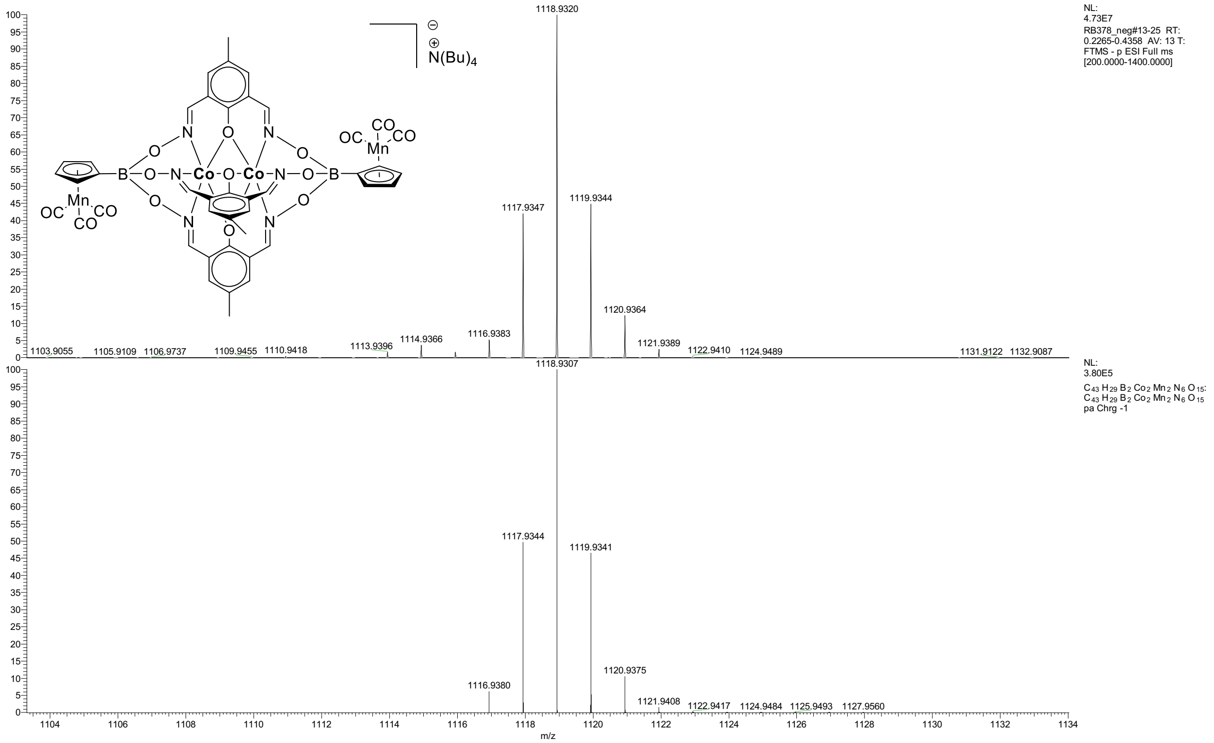

Figure S23. MS (ESI pos, [m/z]; *top*: experimental, *bottom*: simulated) of **5**.

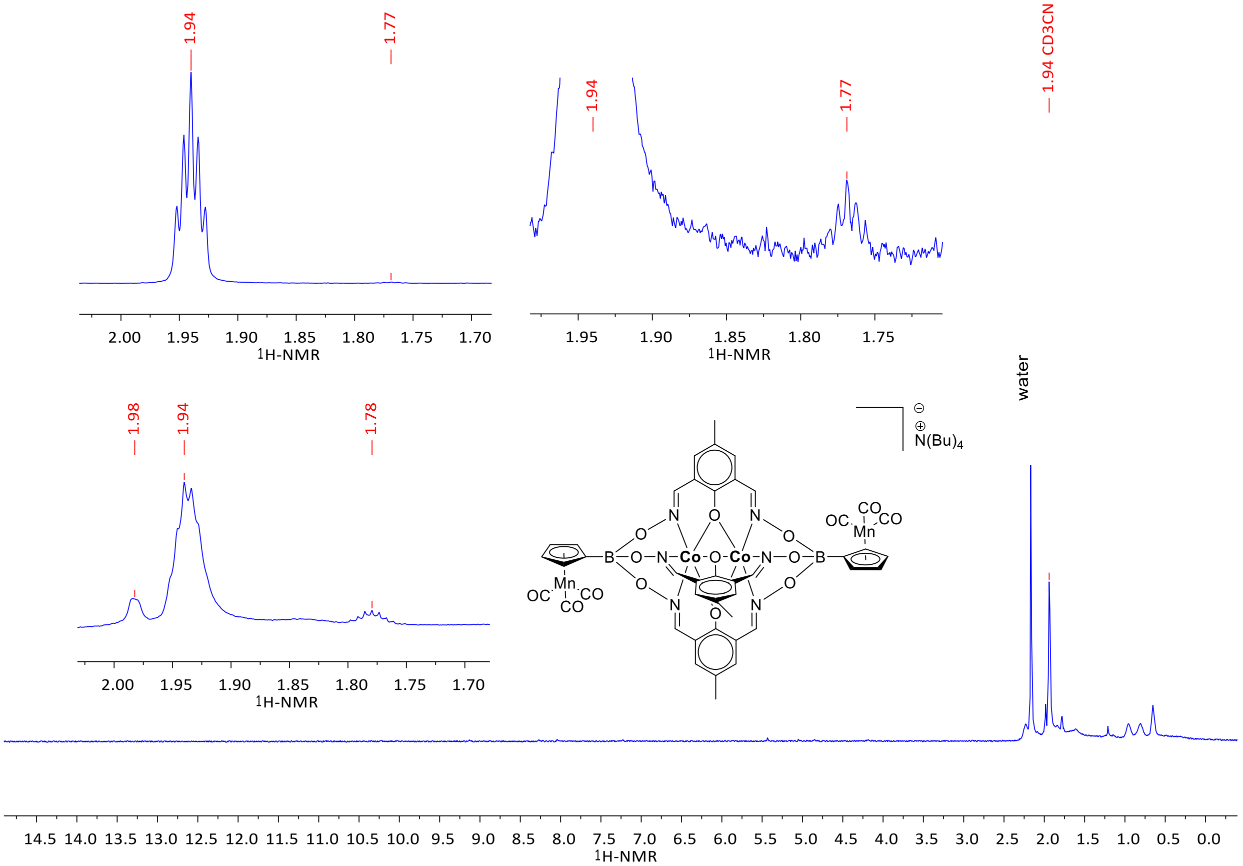

Figure S24. Top: ^{1}H-NMR spectrum of **5**. Bottom: ^{1}H-NMR spectrum of **5** with CD_3CN capillary.

Clathrochelate (6)

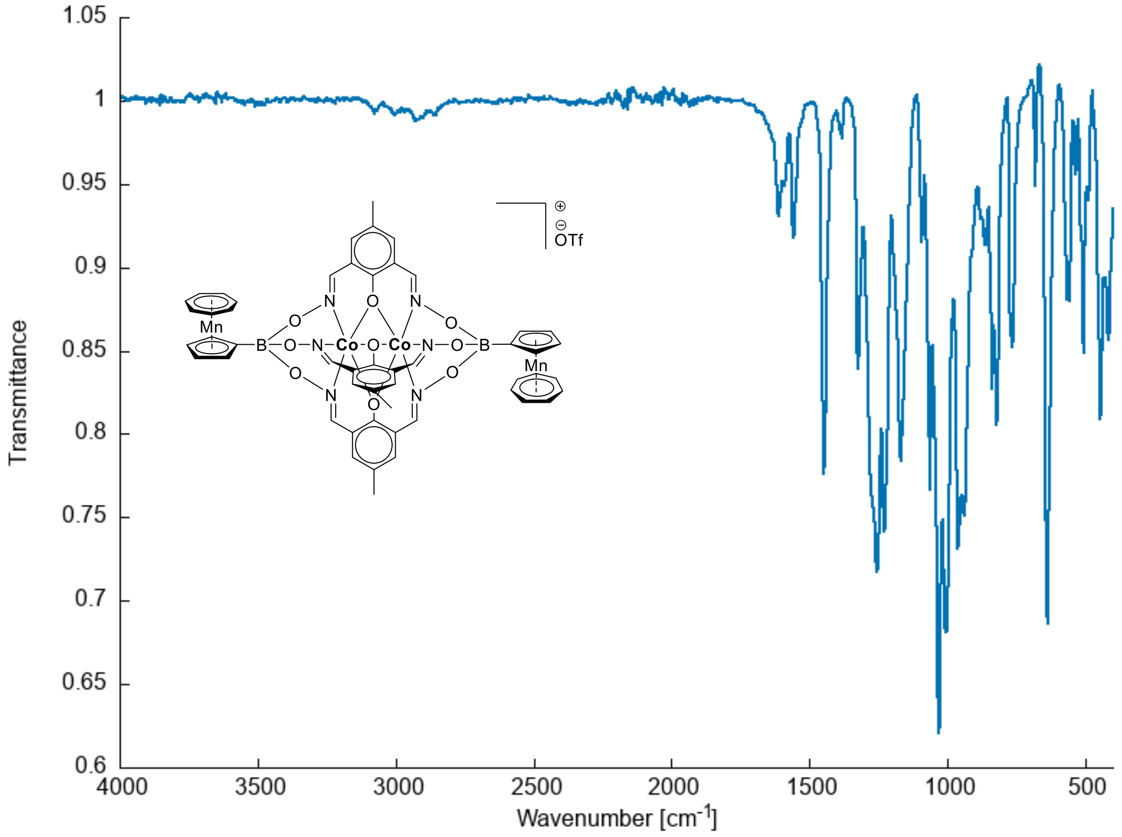

Figure S25. IR spectrum of **6**.

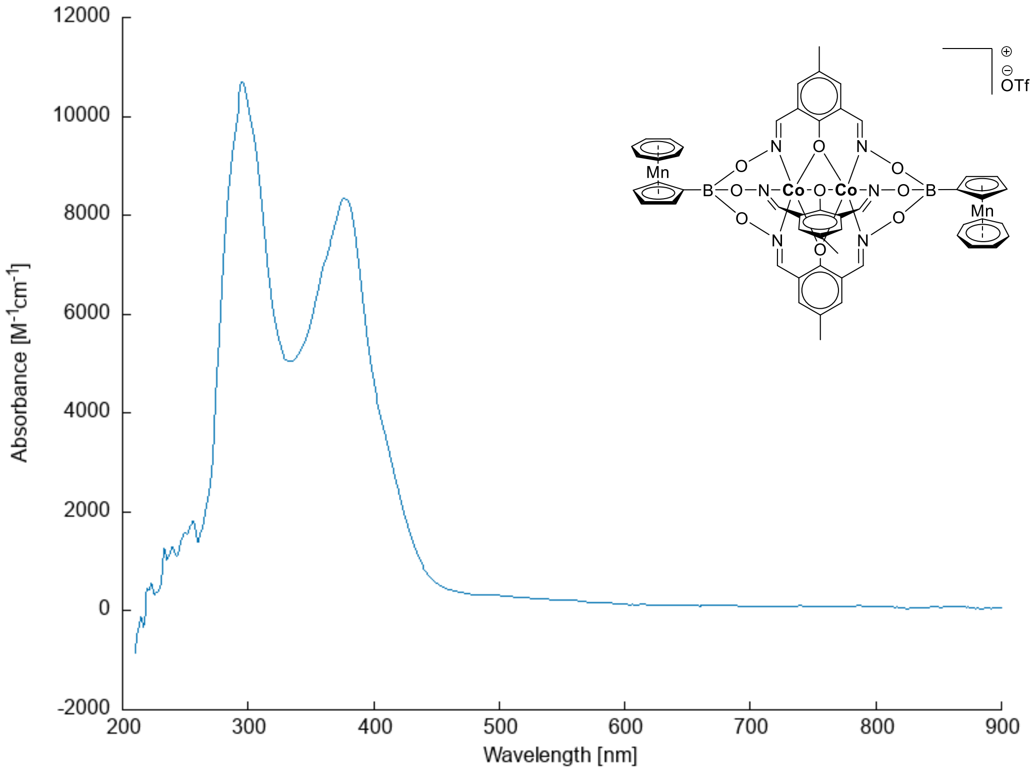

Figure S26. UV/vis spectrum of **6**.

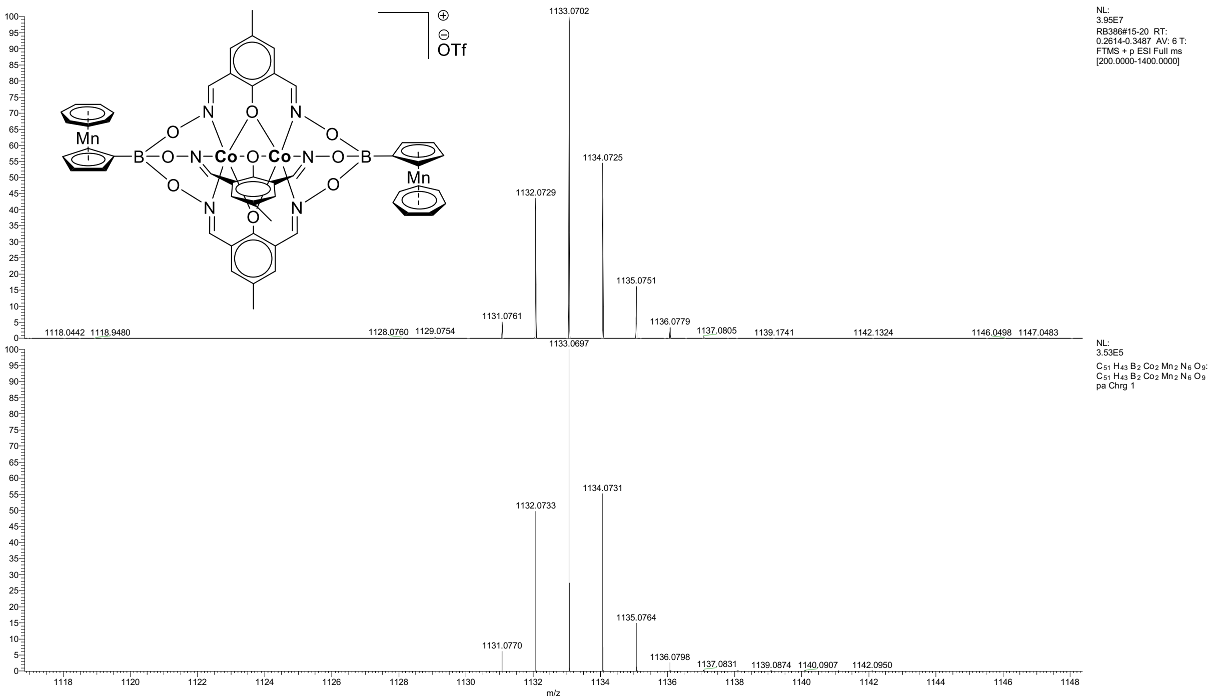

Figure S27. MS (ESI pos, [m/z]; *top*: experimental, *bottom*: simulated) of **6**.

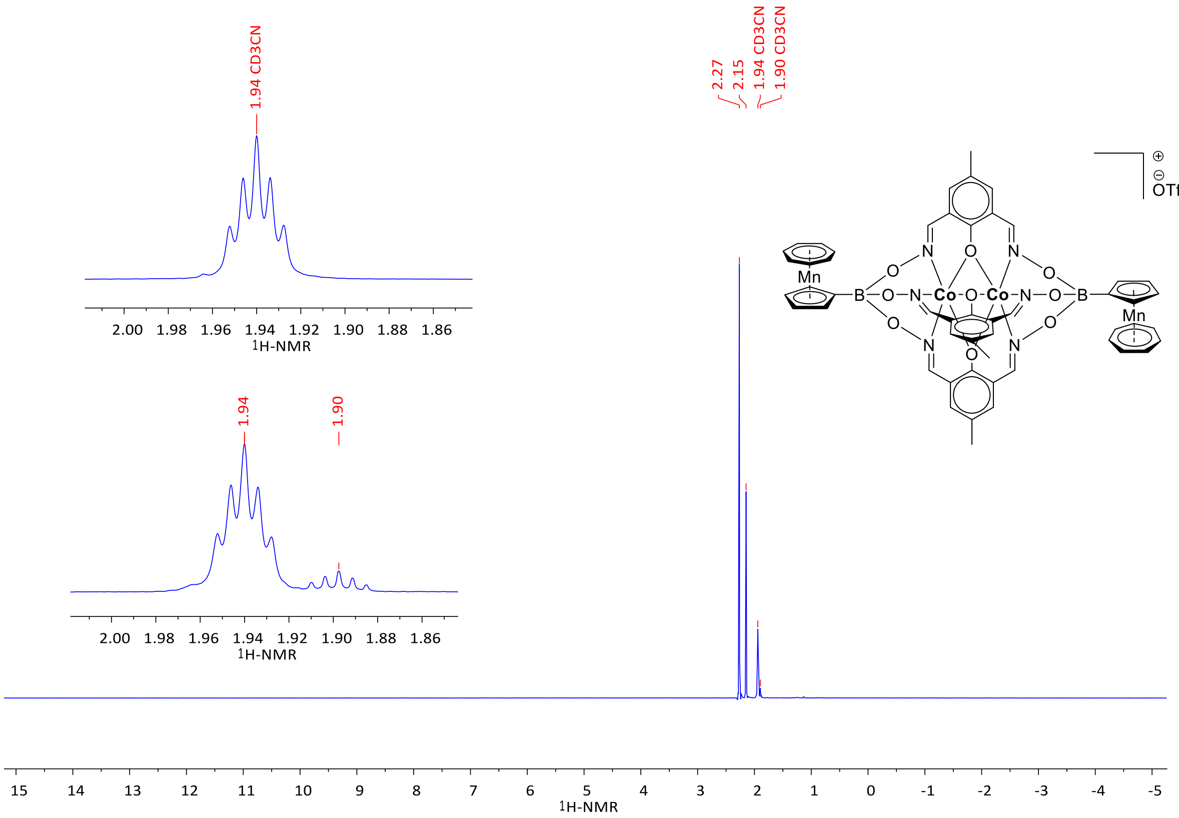

Figure S28. Top: ^{1}H-NMR spectrum of **6**. Bottom: ^{1}H-NMR spectrum of **6** with CD_3CN capillary.

Clathrochelate (7)

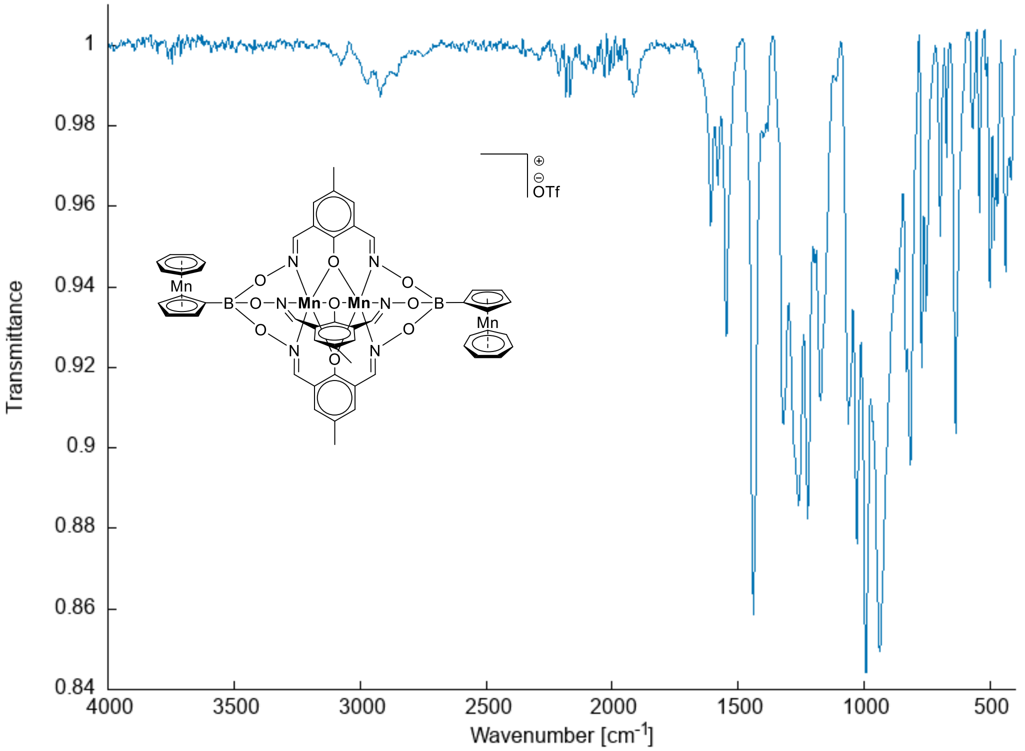

Figure S29. IR spectrum of **7**.

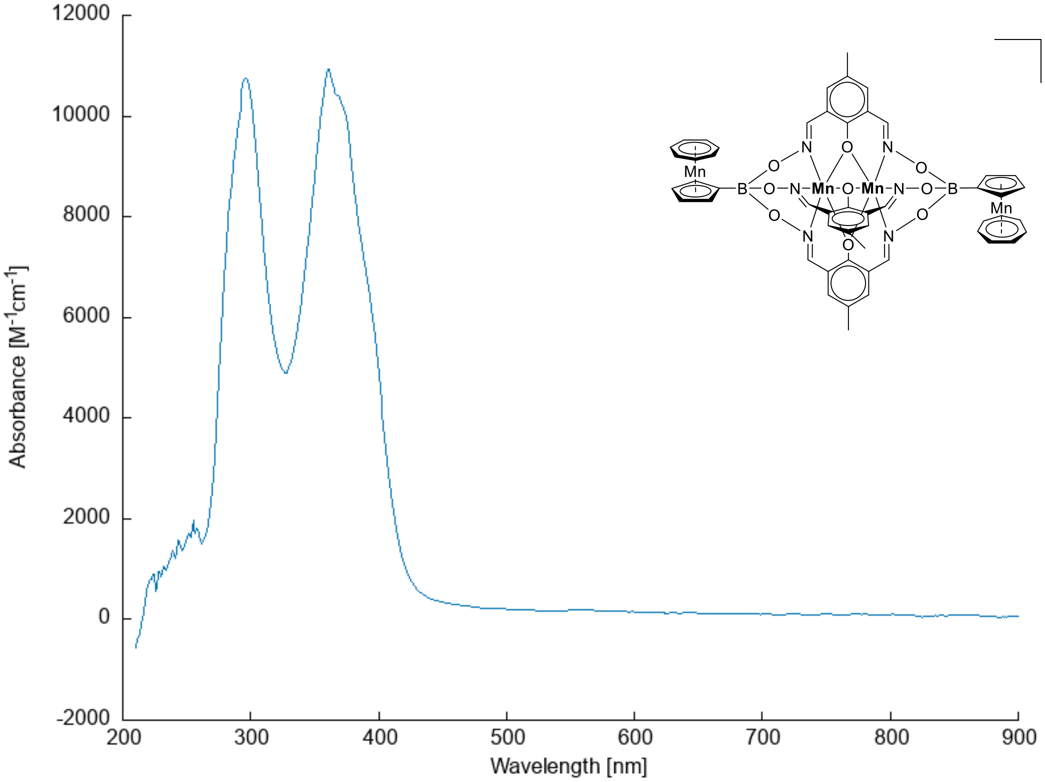

Figure S30. UV/vis spectrum of **7**.

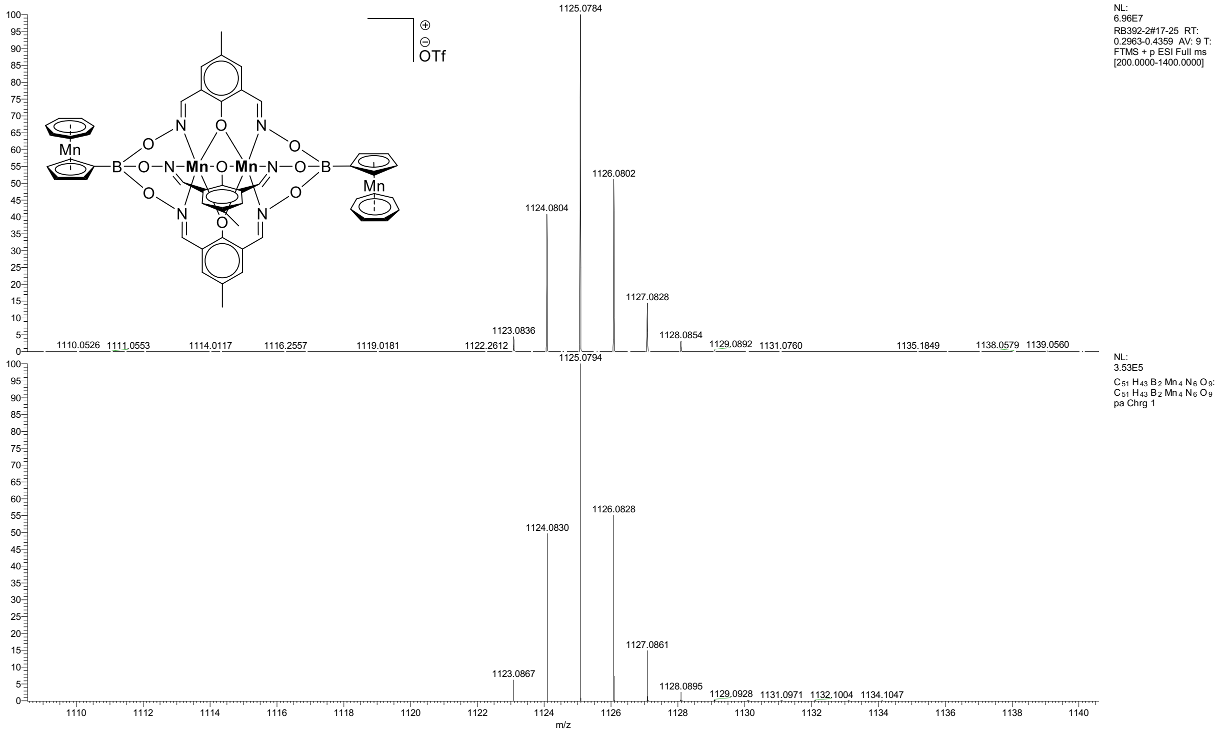

Figure S31. MS (ESI pos, [m/z]; *top*: experimental, *bottom*: simulated) of **7**.

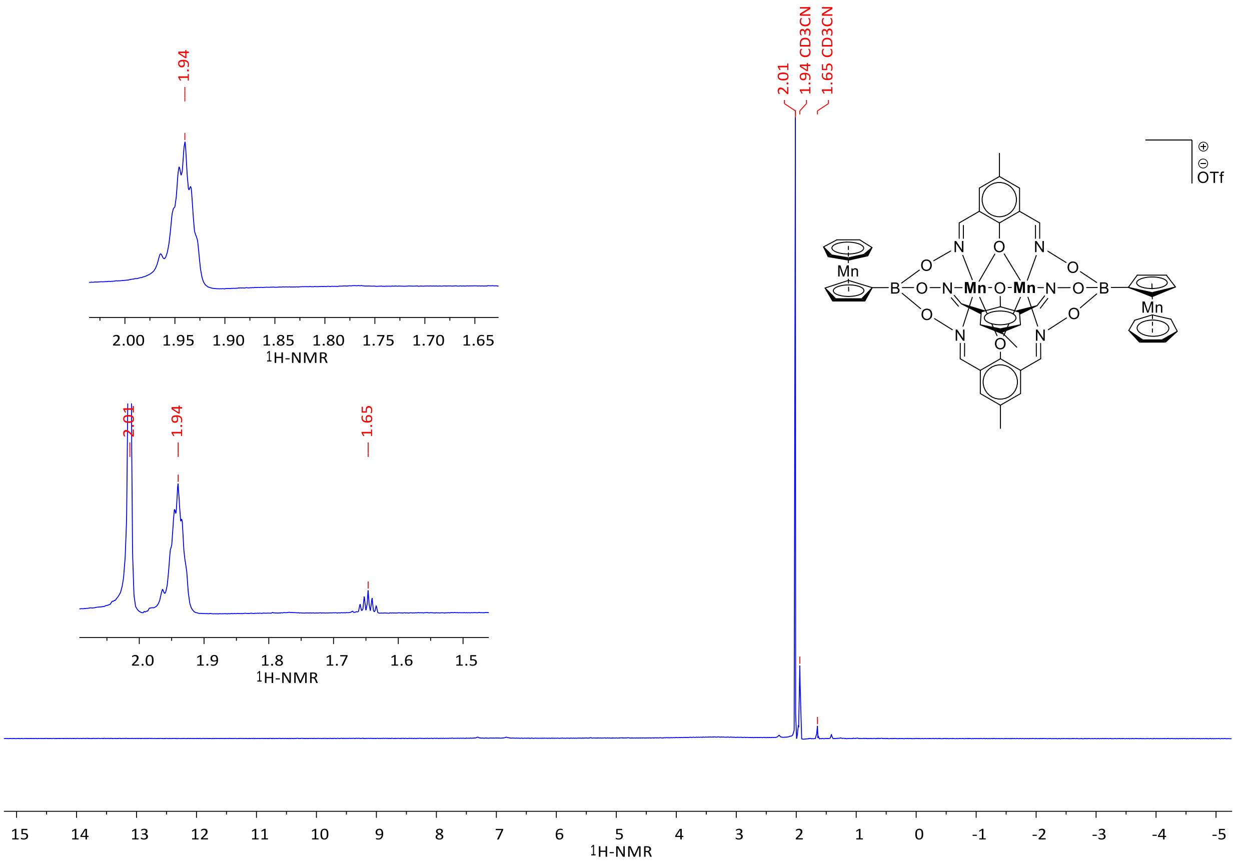

Figure S32. Top: ^{1}H-NMR spectrum of **7**. Bottom: ^{1}H-NMR spectrum of **7** with CD_3CN capillary.

2. Cyclic Voltammetry

Clathrochelate (1)

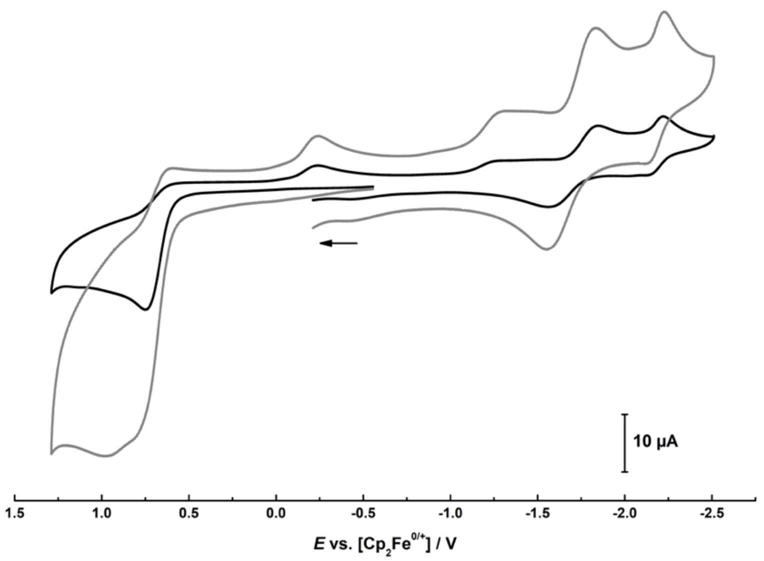

Figure S33. Cyclic voltammogram of **1** in acetonitrile at scan rates of 0.1 Vs^{-1} (black line) and 0.6 Vs^{-1} (gray line).

Clathrochelate (2)

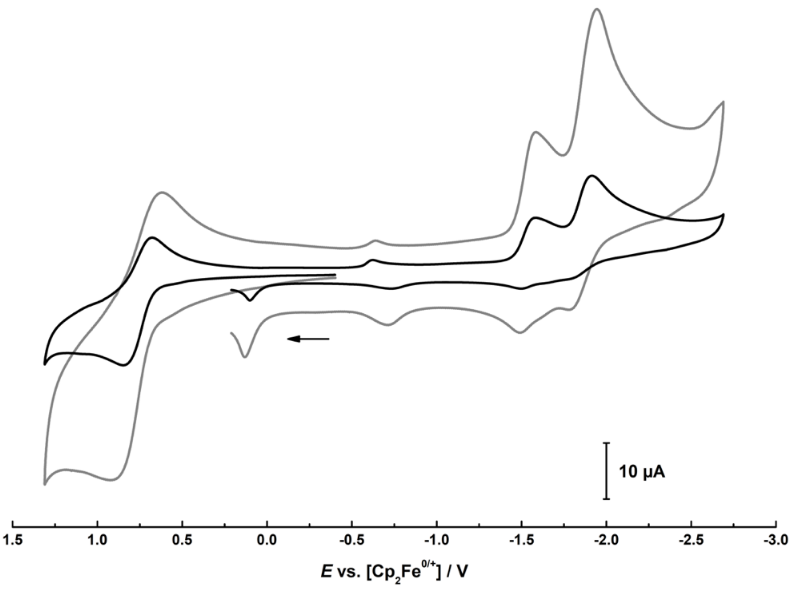

Figure S34. Cyclic voltammogram of **2** in acetonitrile at scan rates of 0.1 Vs^{-1} (black line) and 0.6 Vs^{-1} (gray line).

Clathrochelate (3)

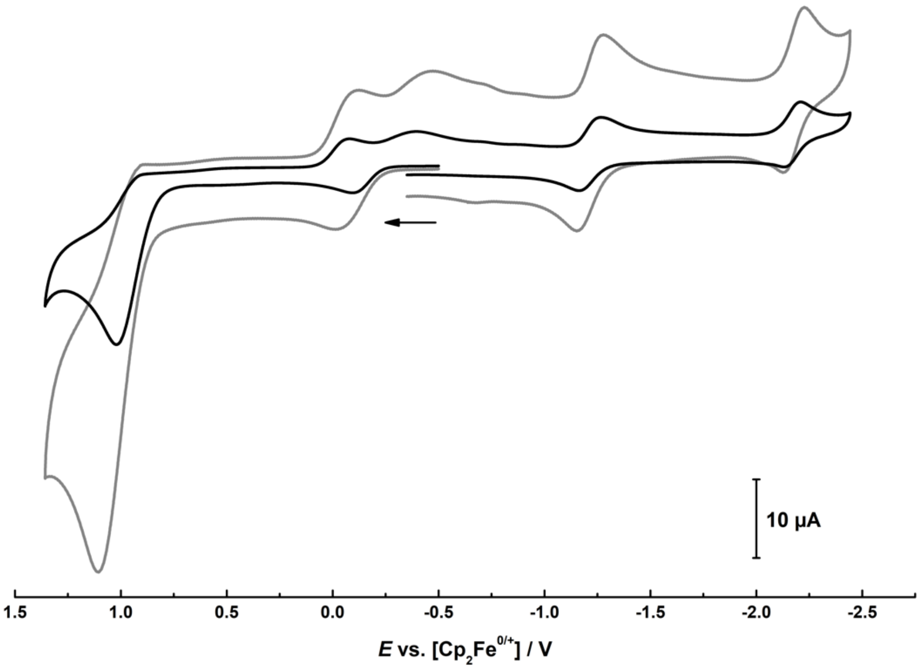

Figure S35. Cyclic voltammogram of **3** in acetonitrile at scan rates of 0.1 Vs^{-1} (black line) and 0.6 Vs^{-1}(gray line).

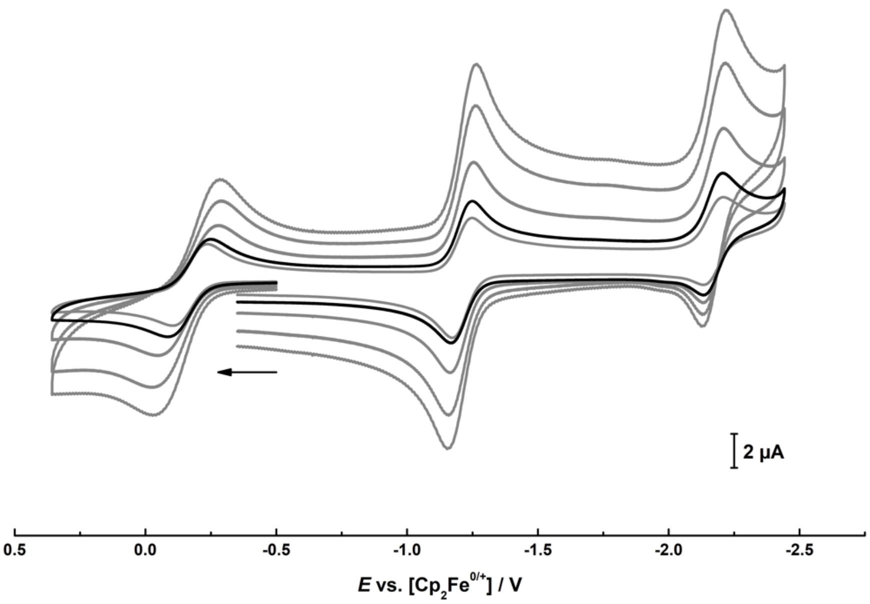

Figure S36. Cyclic voltammogram of **3** in acetonitrile truncated after the first oxidation at sweep rates of 0.05 Vs^{-1}, 0.10 Vs^{-1} (black line), 0.20 Vs^{-1}, 0.40 Vs^{-1}, 0.60 Vs^{-1}.

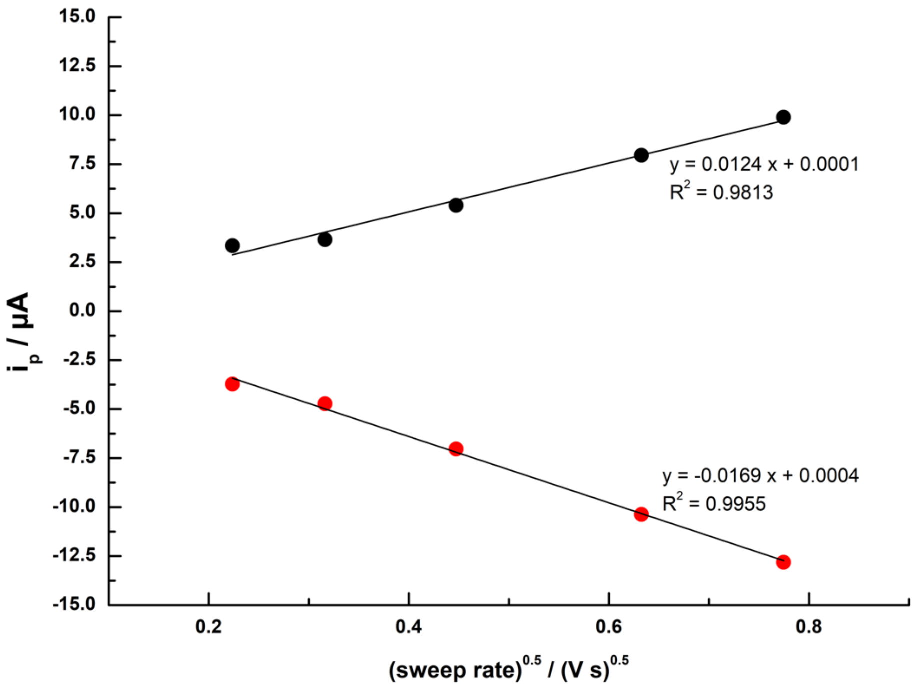

Figure S37. Randles-Sevcik plot of the first quasi-reversible reduction of **3**

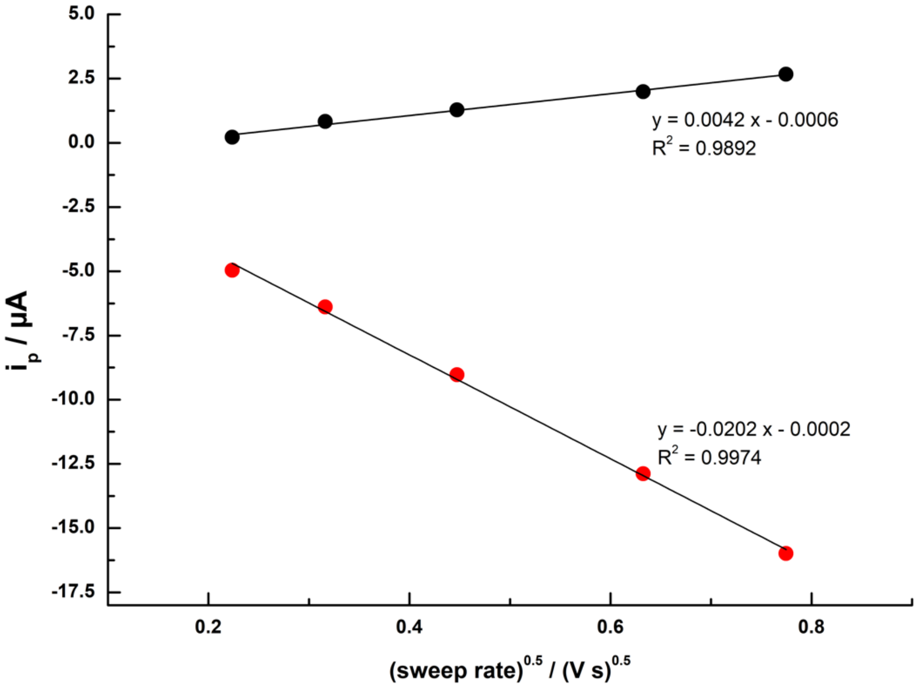

Figure S38. Randles-Sevcik plot of the second irreversible reduction of **3**.

Clathrochelate (4)

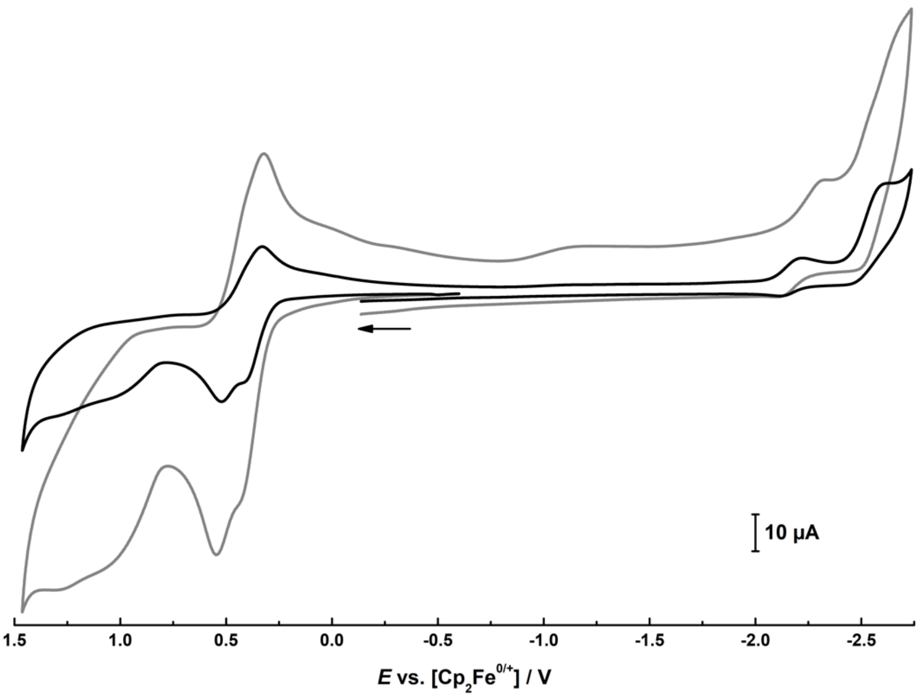

Figure S39. Cyclic voltammogram of **4** in acetonitrile at scan rates of 0.10 Vs^{-1} (black line) and 0.60 Vs^{-1} (gray line).

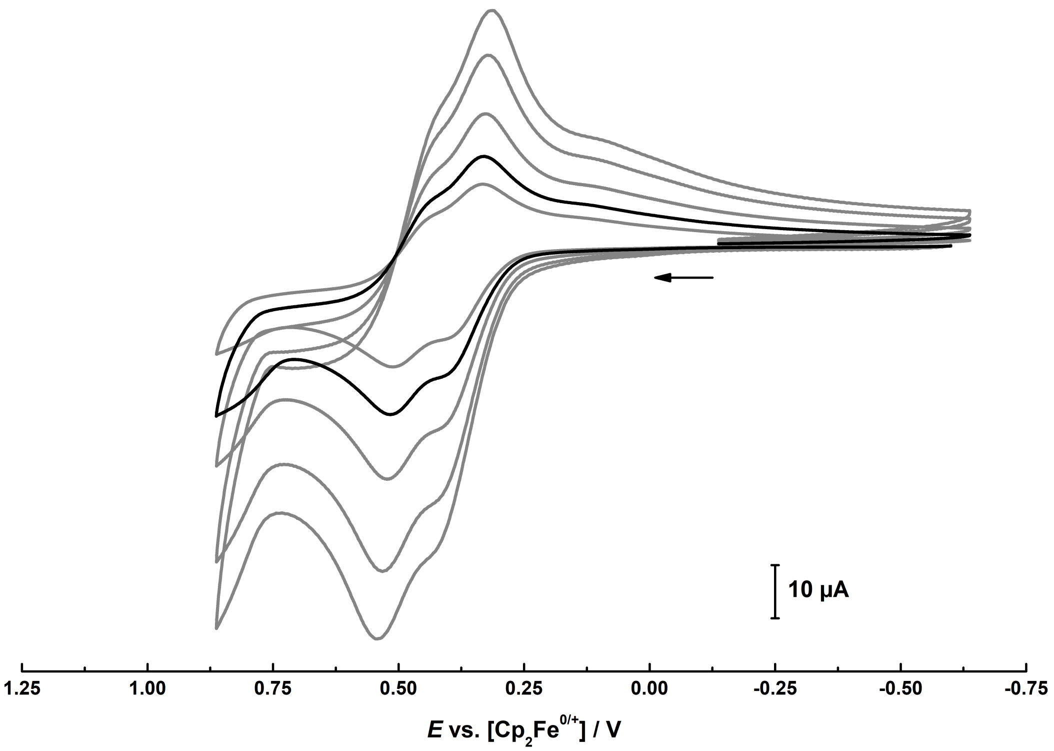

Figure S40. Cyclic voltammogram truncated the first oxidation at sweep rates of 0.05 Vs^{-1}, 0.10 Vs^{-1} (black line), 0.20 Vs^{-1}, 0.40 Vs^{-1}, 0.60 Vs^{-1} of **4**.

Clathrochelate (5)

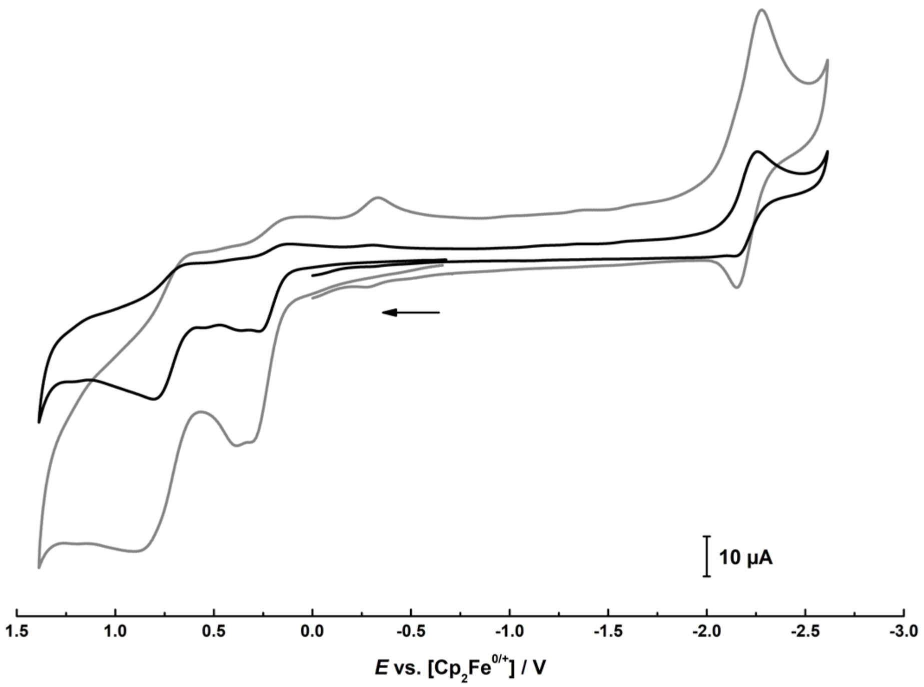

Figure S41. Cyclic voltammogram of **5** in acetonitrile at scan rates of 0.10 Vs^{-1} (black line) and 0.60 Vs^{-1} (gray line).

Clathrochelate (6)

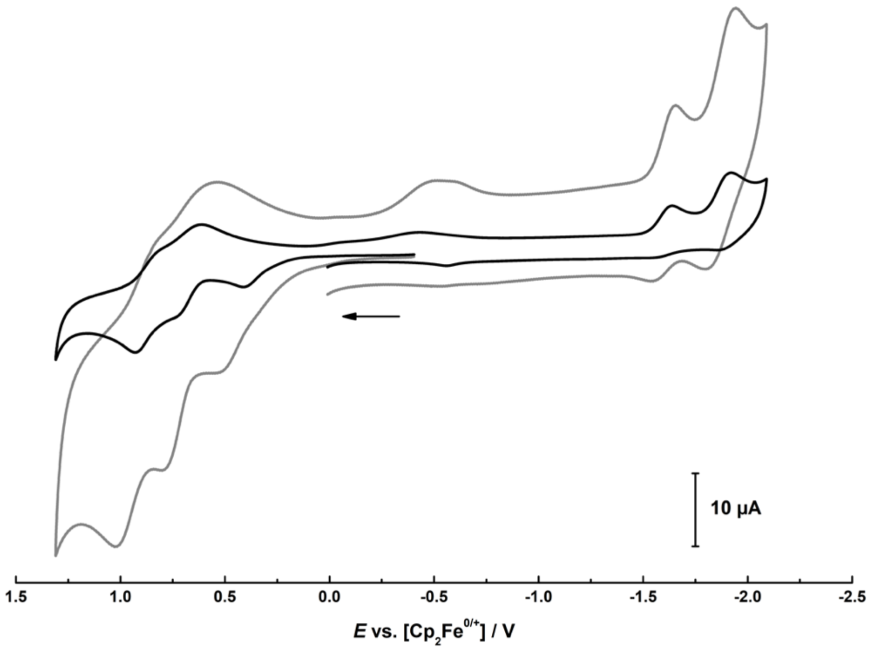

Figure S42. Cyclic voltammogram of **6** in acetonitrile at scan rates of 0.10 Vs^{-1} (black line) and 0.60 Vs^{-1} (gray line).
